# Developing a Machine Learning Model for Predicting 30-Day Major Adverse Cardiac and Cerebrovascular Events in Patients Undergoing Noncardiac Surgery: Retrospective Study

**DOI:** 10.2196/66366

**Published:** 2025-04-09

**Authors:** Ju-Seung Kwun, Houng-Beom Ahn, Si-Hyuck Kang, Sooyoung Yoo, Seok Kim, Wongeun Song, Junho Hyun, Ji Seon Oh, Gakyoung Baek, Jung-Won Suh

**Affiliations:** 1 Cardiovascular Center, Department of Internal Medicine Seoul National University Bundang Hospital Seongnam-si Republic of Korea; 2 Department of Internal Medicine Seoul National University College of Medicine Seoul Republic of Korea; 3 Office of eHealth Research and Businesses Seoul National University Bundang Hospital Seongnam-si Republic of Korea; 4 Department of Health Science and Technology Graduate School of Convergence Science and Technology Seoul National University Seoul Republic of Korea; 5 Division of Cardiology, Department of Internal Medicine Asan Medical Center University of Ulsan College of Medicine Seoul Republic of Korea; 6 Department of Information Medicine Big Data Research Center Asan Medical Center Seoul Republic of Korea; 7 Big Data Research Center Asan Institute for Life Sciences Asan Medical Center Seoul Republic of Korea

**Keywords:** perioperative risk evaluation, noncardiac surgery, prediction models, machine learning, common data model, ML, predictive modeling, cerebrovascular, electronic health records, EHR, clinical practice, risk, noncardiac surgeries, perioperative

## Abstract

**Background:**

Considering that most patients with low or no significant risk factors can safely undergo noncardiac surgery without additional cardiac evaluation, and given the excessive evaluations often performed in patients undergoing intermediate or higher risk noncardiac surgeries, practical preoperative risk assessment tools are essential to reduce unnecessary delays for urgent outpatient services and manage medical costs more efficiently.

**Objective:**

This study aimed to use the Observational Medical Outcomes Partnership Common Data Model to develop a predictive model by applying machine learning algorithms that can effectively predict major adverse cardiac and cerebrovascular events (MACCE) in patients undergoing noncardiac surgery.

**Methods:**

This retrospective observational network study collected data by converting electronic health records into a standardized Observational Medical Outcomes Partnership Common Data Model format. The study was conducted in 2 tertiary hospitals. Data included demographic information, diagnoses, laboratory results, medications, surgical types, and clinical outcomes. A total of 46,225 patients were recruited from Seoul National University Bundang Hospital and 396,424 from Asan Medical Center. We selected patients aged 65 years and older undergoing noncardiac surgeries, excluding cardiac or emergency surgeries, and those with less than 30 days of observation. Using these observational health care data, we developed machine learning–based prediction models using the observational health data sciences and informatics open-source *patient-level prediction* package in R (version 4.1.0; R Foundation for Statistical Computing). A total of 5 machine learning algorithms, including random forest, were developed and validated internally and externally, with performance assessed through the area under the receiver operating characteristic curve (AUROC), the area under the precision-recall curve, and calibration plots.

**Results:**

All machine learning prediction models surpassed the Revised Cardiac Risk Index in MACCE prediction performance (AUROC=0.704). Random forest showed the best results, achieving AUROC values of 0.897 (95% CI 0.883-0.911) internally and 0.817 (95% CI 0.815-0.819) externally, with an area under the precision-recall curve of 0.095. Among 46,225 patients of the Seoul National University Bundang Hospital, MACCE occurred in 4.9% (2256/46,225), including myocardial infarction (907/46,225, 2%) and stroke (799/46,225, 1.7%), while in-hospital mortality was 0.9% (419/46,225). For Asan Medical Center, 6.3% (24,861/396,424) of patients experienced MACCE, with 1.5% (6017/396,424) stroke and 3% (11,875/396,424) in-hospital mortality. Furthermore, the significance of predictors linked to previous diagnoses and laboratory measurements underscored their critical role in effectively predicting perioperative risk.

**Conclusions:**

Our prediction models outperformed the widely used Revised Cardiac Risk Index in predicting MACCE within 30 days after noncardiac surgery, demonstrating superior calibration and generalizability across institutions. Its use can optimize preoperative evaluations, minimize unnecessary testing, and streamline perioperative care, significantly improving patient outcomes and resource use. We anticipate that applying this model to actual electronic health records will benefit clinical practice.

## Introduction

Major adverse cardiac and cerebrovascular events (MACCE) are among the leading causes of perioperative morbidity and mortality following noncardiac surgeries, particularly in an aging population [1-4]. With over 300 million noncardiac surgeries performed annually, accurate preoperative risk assessment has become essential to optimize patient outcomes and reduce health care costs [5,6]. However, the predictive accuracy of traditional assessment tools is not consistently high, and various tools are used at different physicians’ discretion [7].

Traditionally, the Revised Cardiac Risk Index (RCRI), which comprises 6 equally weighted components, is extensively used to mitigate major perioperative cardiac complications owing to its simplicity and relatively high predictability of in-hospital major adverse cardiac events (MACE) or cardiovascular-related death [8]. However, the index developed over 2 decades ago has certain challenges, including limited external validation and reduced precision in vascular surgery [9]. These factors may modestly impact its effectiveness in predicting clinical outcomes following noncardiac surgeries in practical clinical environments [10]. Subsequent predictive tools, such as the American College of Surgeons, National Surgical Quality Improvement Project (NSQIP), and NSQIP Myocardial Infarction or Cardiac Arrest, developed after RCRI, also show strong performance in predicting postoperative MACE. However, these tools pose challenges for clinicians in practical clinical use because they rely on subjective predictors, leading to low interrater reliability [11]. Despite their enhanced predictive accuracy, their application in real-world settings is often constrained by these practical limitations. Given these challenges, our research uses machine learning techniques integrated with the Observational Medical Outcomes Partnership (OMOP) Common Data Model (CDM). Recent advances in machine learning have demonstrated significant potential in addressing these limitations by leveraging large-scale electronic health records (EHRs) to develop predictive models with enhanced accuracy and adaptability. Machine learning algorithms can extract meaningful patterns from high-dimensional datasets, facilitating the identification of key predictors for perioperative risks [12]. Furthermore, the OMOP CDM standardizes diverse observational databases, improving data interoperability and facilitating seamless integration of predictive models across institutions. This standardized framework enhances data sharing and model validation across health care systems, ensuring broader applicability and reliability, as highlighted by Ahmadi et al [13] in their evaluation of OMOP CDM’s transformative potential in harmonizing patient data across institutions [13-16].

Compared with traditional tools like RCRI, our model incorporates a significantly larger number of predictors, allowing for a more precise risk assessment. The OMOP CDM framework further enhances this capability by offering a comprehensive and standardized approach to data integration, addressing the limitations of previous models and ensuring adaptability across diverse clinical environments. Building upon this robust foundation, we developed a machine learning–based prediction model that leverages advanced algorithms to analyze complex patterns within extensive patient datasets. Unlike American College of Surgeons, NSQIP, and NSQIP Myocardial Infarction or Cardiac Arrest, which often rely on subjective inputs and are constrained by interrater variability, our model automates predictor integration, ensuring consistency and practicality in real-world applications [17,18]. Through this approach, we aim to provide a more advanced and precise tool for personalized risk prediction, demonstrating improved performance compared to traditional and contemporary predictive models.

## Methods

### Data Sources

The data sources used in this study were selected and standardized to ensure the integrity and compatibility of the collected information. The EHRs were converted to the OMOP CDM, and source codes were mapped to standard vocabularies, including the Systematized Nomenclature Of Medicine Clinical Terms (SNOMEDCT) [19,20]. Data analysis was conducted using the observational health data sciences and informatics (OHDSI) open-source patient-level prediction (PLP) package, which was purposefully designed for standardized analysis and harmonization with the OMOP CDM. These specialized tools have facilitated efficient data processing and analysis across different datasets within the OHDSI data network, and our study strictly follows the guidelines about machine learning predictive models in biomedical research [21]. This collaborative aspect enhances the comparability and generalizability of the prediction models, making them applicable to diverse health care settings. To address potential data loss or variation during the conversion and mapping process, the PLP package uses a systematic approach to generate training data. Covariates that could not be mapped (concept_ID = 0) are excluded from the input data. Subsequently, a sparse matrix is initialized to represent patient-level covariates and infrequently observed covariates—those with a nonzero frequency below a predefined threshold (default 0.1%)—are excluded to reduce noise. Normalization is then performed by scaling covariates to their maximum observed values, and feature selection techniques are applied to retain only meaningful variables for model training. These steps minimize the impact of unconverted or missing data, ensuring the robustness and reliability of the models.

To develop and evaluate our prediction models, we retrospectively used patient data from 2 tertiary hospitals, Seoul National University Bundang Hospital (SNUBH) and Asan Medical Center (AMC), which are recognized for their substantial CDM datasets. The SNUBH dataset contains data from 46,225 patients who underwent noncardiac surgery between January 2003 and December 2020, and the AMC dataset includes data from 396,424 patients who underwent noncardiac surgery between January 2010 and December 2020. This extensive dataset included a comprehensive array of demographic information and detailed preoperative baseline characteristics, including diagnosis codes, underlying diseases, laboratory test results, medications, type of surgery, and clinical outcomes from the EHR system (Table 1).

**Table 1:** Baseline characteristics.

Characteristics						SNUBH^a^	AMC^b^					*P* value
Number of populations, N						46,225	396,424					
Age, years, mean (SD)						72.9 (5.35)	72.9 (6.08)					.01
**Sex, n (%)**												<.001
	Male					25,573 (55.3)	232,522 (58.7)					
	Female					20,652 (44.7)	163,902 (41.3)					
BMI (kg/m^2^), mean (SD)						23.7 (3.39)	23.3 (3.70)					<.001
**Underlying disease, n (%)**												
	Hypertension					28,641 (62)	216,440 (54.6)					<.001
	Diabetes					12,815 (27.7)	104,269 (26.3)					<.001
	Dyslipidemia					12,078 (26.1)	129,601 (32.7)					<.001
	Congestive heart failure					961 (2.1)	19,613 (4.9)					<.001
	Chronic kidney disease					2588 (5.6)	45,095 (11.4)					<.001
	Cerebrovascular disease					7363 (15.9)	40,628 (10.2)					<.001
	Ischemic heart disease					3939 (8.5)	43,302 (10.9)					<.001
**Preoperative lab results**												
	White blood cell (10^3^/μL), mean (SD)					7.0 (2.64)	7.5 (3.56)					<.001
	Hemoglobin (g/dL), mean (SD)					12.8 (1.89)	11.5 (2.24)					<.001
	Platelet (10^3^/μL), mean (SD)					233.9 (75.86)	214.9 (91.24)					<.001
	Sodium (mmol/L), mean (SD)					139.9 (3.42)	138.2 (4.45)					<.001
	Potassium (mmol/L), mean (SD)					4.3 (0.46)	4.2 (0.52)					<.001
	BUN^c^ (mg/dL), mean (SD)					18.3 (9.67)	23.1 (17.06)					<.001
	Creatinine (mg/dL), mean (SD)					1.1 (0.95)	1.3 (1.43)					<.001
	Creatinine level (≥2 mg/Dl), n (%)					3434 (7.4)	74,594 (18.8)					<.001
	Total cholesterol (mg/dL), mean (SD)					169.0 (41.55)	146.2 (45.78)					<.001
	LDL^d^ (mg/dL), mean (SD)					92.2 (30.90)	91.3 (36.14)					.17
	Albumin (g/dL), mean (SD)					4.0 (0.53)	3.2 (0.71)					<.001
	AST^e^ (IU/L), mean (SD)					27.7 (20.58)	31.7 (30.72)					<.001
	ALT^f^ (IU/L), mean (SD)					24.1 (21.70)	25.4 (28.08)					<.001
	Glucose (mg/dL), mean (SD)					122.2 (43.59)	133.0 (55.15)					<.001
	PT^g^ (INR^h^), mean (SD)					1.0 (0.19)	1.1 (0.31)					<.001
	aPTT^i^ (s), mean (SD)					36.6 (5.83)	31.1 (8.13)					<.001
**Medications, n (%)**												
	Aspirin					11,900 (25.7)	139,029 (35.1)					<.001
	P2Y12 inhibitor					6263 (13.5)	71,436 (18)					<.001
	β-blocker					9678 (20.9)	179,264 (45.2)					<.001
	RAS^j^ inhibitor					12,357 (26.7)	161,016 (40.6)					<.001
	Calcium channel blocker					15,771 (34.1)	227,915 (57.5)					<.001
	Statin					11,734 (25.4)	129,236 (32.6)					<.001
	Insulin treatment					8603 (18.6)	138,392 (34.9)					<.001
**Type of surgery^k^**												
	**Intermediate risk (1%-5%), n (%)**											
			Intraperitoneal: splenectomy, hiatal hernia repair, cholecystectomy		1429 (3.1)				29,072 (7.3)		<.001	
			Carotid symptomatic (CEA^l^ or CAS^m^)		17 (0)				655 (0.2)		<.001	
			Peripheral arterial angioplasty		12 (0)				24,861 (6.3)		<.001	
			Head and neck surgery		2090 (4.5)				60,919 (15.4)		<.001	
			Neurological or orthopedic: major (hip and spine surgery)		3309 (7.2)				16,878 (4.3)		<.001	
			Urological or gynecological: major		243 (0.5)				5350 (1.3)		<.001	
			Renal transplant		23 (0)				2684 (0.7)		<.001	
			Intrathoracic: nonmajor		1596 (3.5)				50,247 (12.7)		<.001	
	**High risk (>5%), n (%)**											
		Aortic and major vascular surgery		2028 (4.4)				53,886 (13.6)		<.001		
		Open lower limb revascularization or amputation or thromboembolectomy		250 (0.5)				7786 (2)		<.001		
		Duodeno-pancreatic surgery		247 (0.5)				7216 (1.8)		<.001		
		Liver section, bile duct surgery		373 (0.8)				19,183 (4.8)		<.001		
		Esophagectomy		75 (0.2)				7795 (2)		<.001		
		Repair of perforated bowel		1557 (3.4)				105,525 (26.6)		<.001		
		Adrenal resection		66 (0.1)				1191 (0.3)		<.001		
		Pneumonectomy		1026 (2.2)				13,751 (3.5)		<.001		
		Pulmonary or liver transplant		27 (0.1)				8656 (2.2)		<.001		
		Unspecified		494 (1.1)				74,067 (18.7)		<.001		
	**Outcome**											
		Myocardial infarction		907 (2)				5603 (1.4)		<.001		
		Cardiac arrest or shock		35 (0.1)				168 (0)		.002		
		Heart failure		308 (0.7)				2310 (0.6)		.03		
		Stroke		799 (1.7)				6017 (1.5)		.001		
		Death (in-hospital)		419 (0.9)				11,875 (3)		<.001		

^a^SNUBH: Seoul National University Bundang Hospital.

^b^AMC: Asan Medical Center.

^c^Blood urea nitrogen.

^d^Low density lipoprotein.

^e^AST: aspartate aminotransferase.

^f^ALT: alanine aminotransferase.

^g^PT: prothrombin time.

^h^INR: international normalized ratio.

^i^aPTT: activated partial thromboplastin time.

^j^RAS: renin-angiotensin system.

^k^The surgery risk type was classified into two types: (1) intermediate and (2) high.

^l^CEA: carotid endarterectomy.

^m^CAS: carotid artery stenting.

### Ethical Considerations

This retrospective, observational network study was conducted by a multidisciplinary team comprising cardiologists, medical informatics specialists, and data scientists. The study received approval from the institutional review boards (IRBs) of SNUBH (IRB number 2208-772-906) and AMC (IRB number 2022-1547). Due to the retrospective study design and the use of deidentified data, the requirement for written informed consent was waived.

### Study Design and Target Cohort

**Figure 1:**
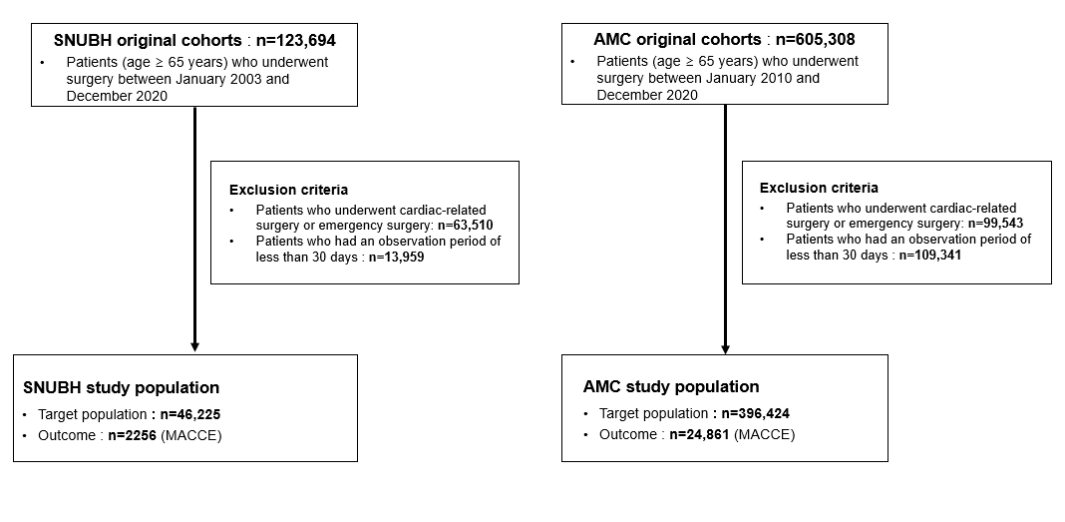
Two tertiary hospital cohort designs. AMC: Asan Medical Center; MACCE: major adverse cardiac and cerebrovascular events; SNUBH: Seoul National University Bundang Hospital.

**Figure 2:**
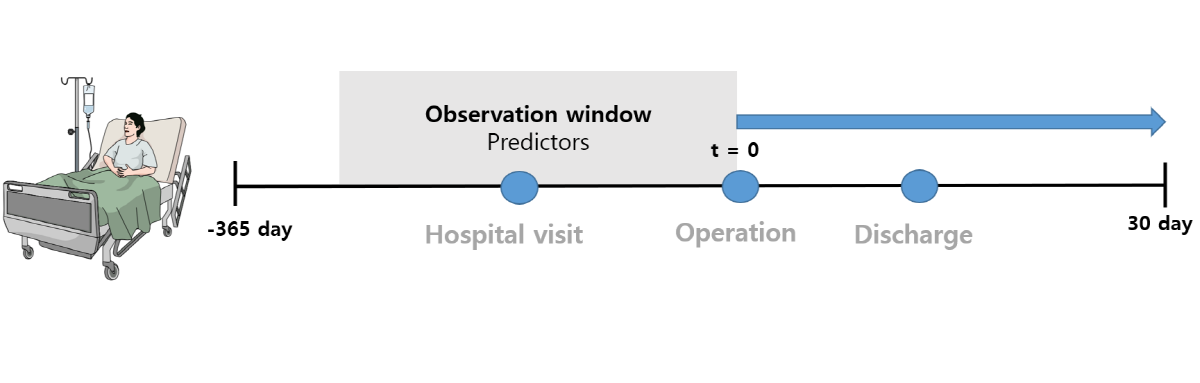
Data collection for predictors.

We conducted a retrospective analysis of patients aged 65 years and older who underwent noncardiac surgeries at 2 independent tertiary hospitals. Age was determined at the time of surgery. We excluded patients who had undergone cardiac or emergency surgery within 3 days of a hospital visit and those who did not have a sufficient observation period of less than 30 days (Figure 1). The prediction time (*t*=0) and start date of the time-at-risk window for prediction were set as surgery dates. The end date of the time-at-risk window for clinical outcomes was 30 days after surgery. The data collection period for the predictors was defined as 3-365 days before the start date of the time-at-risk window (Figure 2). We adjusted the observational time frame to collect baseline characteristics and preoperative laboratory measurements within a narrower window of 3-30 days before the onset of the time-at-risk period. This adjustment ensured that the data accurately represented the patient's condition at the beginning of the time-at-risk period.

### Clinical Outcomes

The clinical outcome of this study was MACCE within 30 days of noncardiac surgery. The individual components of MACCE include myocardial infarction, cardiac arrest or shock, heart failure, stroke, and death. All clinical events were identified and extracted from CDM data using standardized concept IDs (Table S1 in Multimedia Appendix 1). To ensure comprehensive coverage of relevant events, our approach included a broad spectrum of concepts for each MACCE component, ranging from higher-level descriptors to more specific descriptors. Death analysis was based on records from the EHR data within 30 days after noncardiac surgery.

### Prediction Model Development and Validation

Using observational health care data, we used the standardized, open-source OHDSI *PLP* package within R (version 4.1.0; R Foundation for Statistical Computing) to develop and validate our prediction model. We developed a prediction model by integrating data from preoperative laboratory measurements 16 routinely measured basic parameters: white blood cell, hemoglobin, platelet count, aspartate aminotransferase, alanine aminotransferase, blood urea nitrogen, creatinine, albumin, calcium, sodium, phosphate, total bilirubin, c-reactive protein, cholesterol, hemoglobin A_1c_, and prothrombin time), previous diagnosis, medication records, and surgical type from the SNUBH CDM development dataset. This dataset was divided into a training set (34,670/46,225, 75%) and a testing set (11,555/46,225, 25%) for internal validation of the developed model. For the training dataset, we used a 3-fold cross-validation for hyperparameter optimization. Cross-validation was used to minimize overfitting and optimize the model’s generalization capabilities by evaluating its performance on different data splits.

With the OHDSI PLP framework, the least absolute shrinkage and selection operator, logistic regression, gradient boosting machines, AdaBoost, random forest (RF), and decision trees were developed. Model discrimination was assessed using the areas under the receiver operating characteristic curve (AUROC) and areas under the precision-recall curve. In addition, a calibration plot analysis was used to gauge the reliability of the model’s predictions and to confirm that the predicted probabilities matched the actual occurrence probabilities. Finally, the model’s generalizability is evaluated by performing external validation using the AMC CDM dataset. The external validation results demonstrated minimal differences in AUROC values compared to internal validation, confirming the model's ability to generalize effectively to unseen data and supporting the robustness of overfitting prevention strategies such as feature selection, regularization, and cross-validation. To evaluate the relative importance of covariates in developing the prediction model, feature importance was calculated and sorted in descending order based on the most effective machine learning algorithm contributing to the prediction. In addition, we attempted to develop prediction models by recombining variables based on covariate grouping. These recombination models were developed by excluding certain covariate groups with the expectation that this selective process may enhance prediction accuracy by reducing noise and focusing on the most significant factors.

## Results

### Study Population

A total of 46,225 patients were enrolled at SNUBH, and 396,424 were enrolled at AMC, with an average age of 72.9 (SD 5.35) years at both hospitals (Table 1). More male than female patients were enrolled at both institutions, with 25,573/46,225 males (55.3%) at SNUBH and 232,522/396,424 males (58.7%) at AMC. Hypertension was the most common comorbidity, affecting 28,641/46,225 (62%) and 216,440/396,424 (54.6%) of patients at SNUBH and AMC, respectively. At SNUBH, cerebrovascular disease was more common (7363/46,225; 15.9%) compared with 40,628 of 396,424 (10.2%) at AMC. By contrast, congestive heart failure was more common at AMC (19,613/396,424, 4.9%) than at SNUBH (961/46,225, 2.1%). Similarly, ischemic heart disease had a higher representation at AMC (43,302/396,424, 10.9%) than at SNUBH (3939/46,225, 8.5%). Preoperative laboratory results were within normal ranges, with patients with a creatinine level of 2 mg/dL or higher accounting for 3434 of 46,225 (7.4%) at SNUBH and 74,594 of 396,424 (18.8%) at AMC. Regarding medications, the AMC data showed a higher proportion of patients registered with aspirin, P2Y12 inhibitors, beta-blockers, renin-angiotensin system inhibitors, calcium channel blockers, statins, and insulin treatment.

There was a significant difference between the 2 hospitals regarding the type of surgery and post-noncardiac surgery MACCE within 30 days across all categories. Surgeries with a risk exceeding 1% are presented in Table 1; those with unmapped names were classified as unspecified. Post-noncardiac surgery MACCE within 30 days included myocardial infarction, which occurred in 907 of 46,225 (2%) patients at SNUBH and 5603 of 396,424 (1.4%) at AMC, heart failure in 308 of 46,225 (0.7%) and 2310 of 396,424 (0.6%), and strokes in 799 of 46,225 (1.7%) and 6017 of 396,424 (1.5%), respectively. In-hospital deaths accounted for 419 of 46,225 (0.9%) and 11,875 of 396,424 (3%) deaths at SNUBH and AMC, respectively.

### Prediction Model Performance

**Figure 3:**
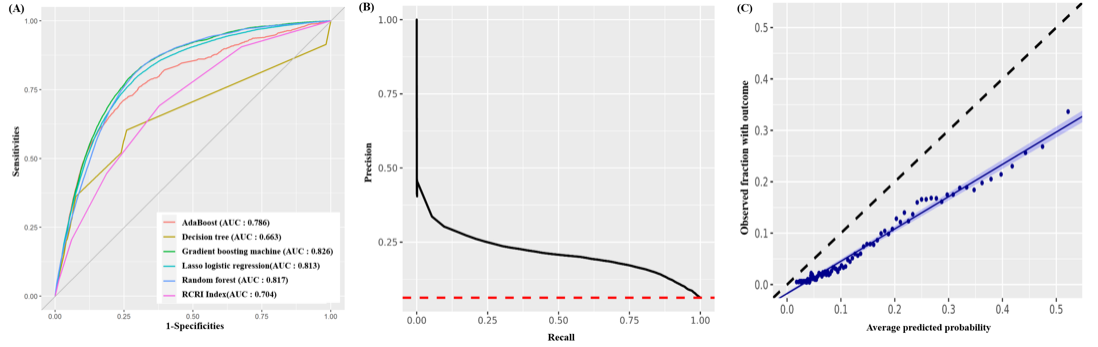
Seoul National University Bundang Hospital (SNUBH) prediction model based on validation data. AUC: area under the curve; RCRI: Revised Cardiac Risk Index.

The predictability of prediction models for internal and external validation is presented in Table 2. The numbers of patients included in the training, test, and external validation sets of the SNUBH model who met the inclusion criteria are presented in Table S2 (Multimedia Appendix 1). When assessed using the RCRI score and compared with 5 other machine learning prediction models, all machine learning models outperformed the RCRI model with a higher AUROC for MACCE prediction than the RCRI score (AUROC 0.704; Figure 3A). The RF generally showed the best overall performance in internal and external validations across outcomes with moderate calibration among the 5 predictive models. The AUROC of this model was 0.897 (0.883-0.911) and 0.817 (0.815-0.819) for internal and external validations, respectively (Figure 3A and Table 2), and the area under the precision-recall curve was 0.095 (Figure 3B). In addition, it demonstrated outstanding calibration, showing strong alignment with the average predicted probability on the calibration plot (Figure 3C).

**Table 2:** Predictability of 5 machine learning prediction models.

Prediction model		SNUBH^a^		AMC^b^
AUROC^c^ (95% CI)		Train	Test	External validation
**MACCE^d^**				
	Random forest	0.985 (0.982-0.989)	0.897 (0.883-0.911)	0.817 (0.815-0.819)
	Gradient boosting machine	0.935 (0.928-0.941)	0.898 (0.885-0.912)	0.826 (0.823-0.828)
	Lasso logistic regression	0.906 (0.899-0.914)	0.892 (0.878-0.906)	0.813 (0.810-0.815)
	AdaBoost	0.907 (0.901-0.914)	0.887 (0.873-0.902)	0.786 (0.782-0.788)
	Decision tree	0.895 (0.885-0.904)	0.776 (0.750-0803)	0.663 (0.659-0.667)

^a^SNUBH: Seoul National University Bundang Hospital.

^b^AMC: Asan Medical Center.

^c^AUROC: area under the receiver operating characteristic curve.

^d^MACCE: major adverse cardiac and cerebrovascular events.

The superior performance of the RF model can be attributed to its unique horizontal ensemble structure that uses bagging to construct decision trees based on randomly selected subsets of features at each split. This structure minimizes tree correlation, reduces overfitting, and handles high-dimensional low-sample size datasets, which are characteristic of electronic medical record data. Furthermore, RF is robust to imbalanced data, outperforming models like gradient boosting machines in scenarios with severe class imbalance. Gradient boosting machines, in contrast, use a boosting structure that sequentially trains weak learners, making them sensitive to noise and rare events and highly dependent on optimal hyperparameter tuning. Compared with simpler models like logistic regression and LASSO, RF excels in capturing complex patterns in high-dimensional data with many irrelevant or noisy features, making it particularly suitable for electronic medical record datasets [13,22-24].

### Predictors

**Figure 4:**
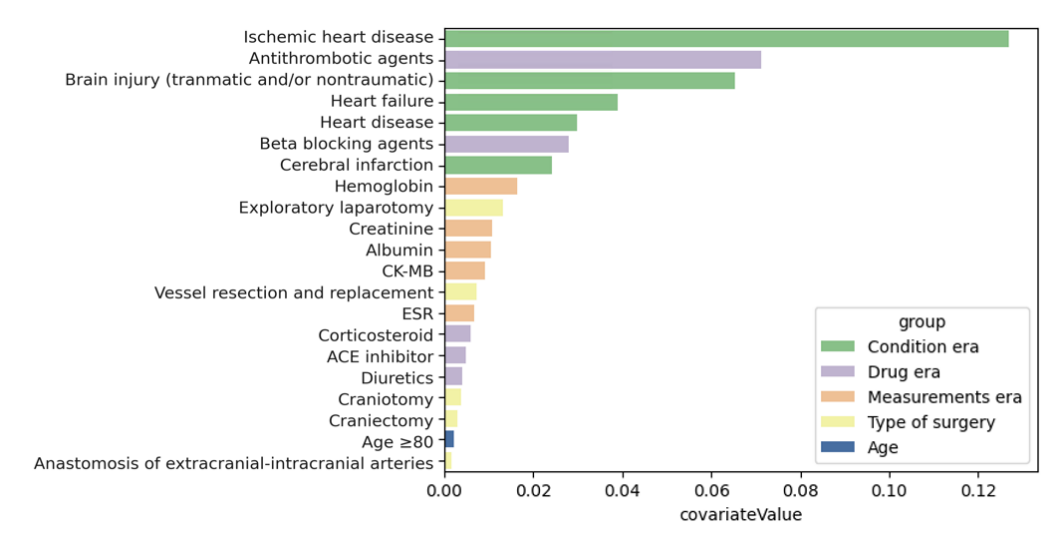
Importance of covariates in the prediction model. CK-MB: creatine kinase-MB; ESR: erythrocyte sedimentation rate.

In the prediction model, we assessed the relative importance of various covariates based on their values (Figure 4). Rather than identifying a single outstanding covariate, the analysis grouped covariates into similar thematic clusters. Predominantly, predictors associated with the patient’s underlying medical history were relatively high in the developed prediction model. These include ischemic heart disease, traumatic and nontraumatic brain injury, heart failure, heart disease, and cerebral infarction. The model highlights the importance of the measurement predictors. Preoperative laboratory measurements revealed that hemoglobin, creatinine, albumin, CK-MB, and erythrocyte sedimentation rates played crucial roles. Among the medication predictors, antithrombotic agents and beta-blockers were notably prominent, whereas the significance of the others was less pronounced. Furthermore, although important, the significance of the type of surgery did not appear to be as substantial as expected when compared with other factors in the model.

In addition, we developed prediction models by recombining the data and considering previous diagnoses, medication, type of surgery, and measurement data in various combinations (Table S3 in Multimedia Appendix 1). However, none of the additional recombination models outperformed the original models. Nevertheless, these models generally exhibited superior predictability compared with RCRI, except for the recombination model that excluded the previous diagnosis group, which yielded results comparable to or slightly inferior to those of RCRI (Figure S1A-C in Multimedia Appendix 1).

## Discussion

### Principal Findings

In this study, we developed and evaluated an advanced perioperative risk prediction model using a CDM-based machine learning approach. The results demonstrated that machine learning models consistently outperformed traditional methods, such as the RCRI score, in predictive accuracy. For instance, the RCRI score achieved an AUROC of 0.704, whereas the RF model, among 5 tested machine learning models, showed the best overall performance with an AUROC of 0.897 for internal validation and 0.817 for external validation. These findings highlight the robustness and generalizability of the model across diverse datasets and outcomes.

This study provides key insights into the potential of CDM-based machine learning to enhance clinical predictive modeling. By achieving superior predictive accuracy and scalability, especially in external validation, our approach demonstrates a promising pathway for developing reliable tools for perioperative risk prediction across institutions. Advances in machine learning for extensive dataset analysis have led to increased interest in applying PLP and offer the potential for medical practice to consider personalized risks as part of clinical decision-making [25]. The adoption of the OMOP CDM has streamlined the transformation of diverse concept domains, encompassing medical conditions, drugs, procedures, and measurements derived from health record systems or reported information into labeled analytic data. This transformation ensures semantic and syntactic interoperability, enhancing the extraction of prediction variables and facilitating seamless integration across various health care data sources [19,26]. The expanding adoption of the OMOP CDM across health care institutions globally further strengthens the transferability of predictive models. For example, over 60 databases in South Korea, covering approximately 73 million patients, have been converted to the OMOP CDM format. This widespread implementation promotes interoperability, cross-institutional research, and scalability of predictive models in diverse health care environments. In addition, the standardized data across different institutions allowed a fair evaluation of the predictive performance of the models by extensive external validation [18]. To implement this framework, we used the OHDSI “Patient-Level Prediction” package, which integrates seamlessly with the OMOP CDM and offers significant advantages. This package not only ensures model reproducibility and transparency through its open-source nature but also provides flexibility in choosing machine learning algorithms and feature engineering techniques. Furthermore, its capability for internal and external validation aligns with best practices, promoting robust performance evaluation and generalizability [20]. Therefore, our model supports existing preoperative evaluation guidelines and enables open dissemination that can be extensively validated across OHDSI collaborator networks.

The well-structured and labeled dataset improves algorithms in supervised machine learning but sometimes leads to overfitting, which prevents the model’s generalization to fit the observed data well [27,28]. To overcome the challenge of overfitting, we used a feature selection method as one of several techniques to identify and prioritize factors essential for the learning process [29,30]. In addition, we used a feature selection method to create new combinations of thematic clusters, including medical conditions, drugs, types of procedures, and measurements, to assess their relative importance in predicting adverse outcomes following surgery. The recombination model, which included past medical conditions and previous laboratory data, exhibited a notably high predictive accuracy. Our model’s ability to discern the varying importance of these factors in real clinical contexts underscores the importance of focusing on patient histories and prior laboratory results during preoperative evaluations [31]. This approach aligns with physicians’ subjective assessments in clinical settings and provides a flexible alternative to traditional methods that may not fully accommodate each patient’s unique circumstances [32].

The practical implications of our research extend to potential time and cost savings in clinical settings. Risk assessments often lead to unnecessary procedures or examinations, such as echocardiography, cardiac computed tomography, and cardiac stress tests, being performed on patients [33]. These tests, even when not closely associated with the patient’s postsurgical outcomes, contribute to ongoing wastage in overall medical costs [34-36]. Several studies have demonstrated that predictive models can effectively reduce unnecessary preoperative testing and associated costs. For example, standardized preoperative models have shown significant reductions in coagulation and renal panel tests, leading to improved resource use without compromising patient safety. Similarly, machine learning–based tools, such as MySurgeryRisk (Azra Bihorac, University of Florida), have enhanced risk stratification for postoperative complications, thereby minimizing the need for unnecessary evaluations. These findings highlight the potential of predictive models to address inefficiencies and optimize preoperative care. Our model, with its high predictive accuracy, is poised to reduce the number of unnecessary tests performed and contribute to medical cost savings. Furthermore, our model could reduce waiting times for patients as unnecessary consultations and tests may be minimized, ultimately mitigating the challenges posed by health care system congestion and assisting patients in undergoing surgery at an appropriate time. In the future, with precise preoperative predictability, we plan to use our model to proactively identify individuals at risk of postsurgical complications and ensure appropriate postoperative management. In an aging population, where surgical mortality and morbidity rates are increasing [37], this approach can serve as a viable solution to effectively mitigate these challenges.

### Strengths

By using the OMOP CDM framework for data standardization, we ensured syntactic and semantic interoperability across diverse datasets. Despite the inherent inconsistencies and missing values often observed in large health care datasets, we mitigated these issues by leveraging data from 2 of the largest tertiary hospitals in South Korea, where the data quality and quantity were sufficient to minimize noise and missing data. This rich dataset enabled the development of a robust machine learning model with high predictive accuracy for perioperative risk assessment.

Furthermore, external validation using datasets from independent institutions demonstrated minimal performance differences compared with internal validation, suggesting that the semantic gap was relatively small. This highlights the model’s strong generalizability and supports its applicability across multiple hospitals. These findings align with previous research emphasizing the importance of model calibration for diverse clinical settings and the need for strategies that address cross-institutional variability without requiring site-specific data harmonization [12,38].

In addition, the model consistently outperformed traditional tools, such as the RCRI score, achieving high AUROC values in both internal and external validations. The inclusion of comprehensive preoperative variables, including laboratory results, medications, and comorbidities, provided a personalized approach to risk prediction. This capability has the potential to reduce unnecessary preoperative testing, streamline clinical decision-making, and improve resource allocation in real-world health care settings.

### Limitations

This study has several limitations. First, the use of datasets from 2 tertiary hospitals introduced variability in patient populations and clinical practices, which could have influenced model performance. However, this diversity reflects real-world conditions and likely contributed to the model’s robustness, as demonstrated by consistent results in external validation. Second, although the model achieved high AUROC values, the relatively low incidence rate suggests challenges in handling imbalanced outcomes. Feature selection was used to prioritize significant predictors, and future studies may incorporate advanced techniques to address this limitation. Third, the model has not yet been tested in real-time clinical workflows, where factors such as delayed data entry could impact performance. The use of standardized OMOP CDM data ensures scalability and future studies will focus on prospective validation in live clinical settings. Finally, the exclusion of certain covariates, such as frailty scores and socioeconomic status, may have limited the model’s predictive accuracy. To maintain scalability across institutions, the study prioritized universally available predictors, but future work will explore integrating additional variables to enhance performance.

### Conclusions

In this study, we successfully developed a high-performance machine learning–based preoperative prediction model by using the standardized data format of the OMOP CDM. This approach offers the potential for improved clinical decision-making and extensive external validation across health care institutions. In the future, our research has practical implications for potential time and cost savings in clinical settings by reducing unnecessary procedures, tests, and consultations, ultimately addressing health care system congestion and improving patient surgical timing.

## Appendix

Multimedia Appendix 1 Supplementary tables and figures.

## Abbreviations

AMC: Asan Medical Center
AUROC: area under the receiver operating characteristic
CDM: Common Data Model
EHR: electronic health record
IRB: institutional review board
MACCE: major adverse cardiac and cerebrovascular events
MACE: major adverse cardiac events
NSQIP: National Surgical Quality Improvement Project
OHDSI: observational health data sciences and informatics
OMOP: Observational Medical Outcomes Partnership
PLP: patient-level prediction
RCRI: Revised Cardiac Risk Index
RF: random forest
SNOMEDCT: systematized nomenclature of medicine clinical terms
SNUBH: Seoul National University Bundang Hospital

## Data Availability

The datasets generated during this study are not publicly available due to data protection agreements but are available from the corresponding author on reasonable request.
