# Supplementary material for: Developing a Machine Learning Model for Predicting 30-Day Major Adverse Cardiac and Cerebrovascular Events in Patients Undergoing Noncardiac Surgery: Retrospective Study
Source: J Med Internet Res. 2025 Apr 9;27:e66366. doi: 10.2196/66366 (PMC12018863; doi:10.2196/66366)
Supplement: Multimedia Appendix 1 [file jmir_v27i1e66366_app1.docx]

**Table S1. Train, test and validation cohorts in two tertiary hospitals**

|  | **SNUBH** | | **AMC** |
| --- | --- | --- | --- |
|  | **Train** | **Test** | **External**  **Validation** |
| **Sample size (meet inclusion criteria)** | **34,670** | **11,555** | **396,424** |
| **MACCE (n, %)** | **1692(4.9%)** | **564(4.9%)** | **24,853(6.3%)** |

**Table S2. Internal and external validations of extra-combination prediction models.**

|  | **Demographic** | **Measurement** | **Underlying history** | **Type of surgery** | **Medication** |
| --- | --- | --- | --- | --- | --- |
| **Prediction model** | **O** | **O** | **O** | **O** | **O** |
| **Recombination (1)** | **O** | **O** | **O** | **O** | **X** |
| **Recombination (2)** | **O** | **O** | **O** | **X** | **O** |
| **Recombination (3)** | **O** | **O** | **X** | **O** | **X** |

| **Recombination (1)** | SNUBH | | AMC |
| --- | --- | --- | --- |
| AUROC (95% CI) | Train | Test | External  Validation |
| **MACCE** | | | |
| Random forest | 0.975 (0.971-0.979) | 0.896 (0.883-0.910) | 0.814 (0.812-0.817) |
| Gradient boosting machine | 0.917 (0.910-0.925) | 0.896 (0.882-0.910) | 0.821 (0.818-0.823) |
| Lasso Logistic Regression | 0.905 (0.897-0.912) | 0.894 (0.880-0.907) | 0.792 (0.789-0.795) |
| AdaBoost | 0.906 (0.899-0.912) | 0.881 (0.866-0.897) | 0.756 (0.752-0.759) |
| DecisionTree | 0.894 (0.885-0.903) | 0.820 (0.797-0.843) | 0.708 (0.705-0.712) |

| **Recombination (1)** | AMC | | SNUBH |
| --- | --- | --- | --- |
| AUROC (95% CI) | Train | Test | External  Validation |
| **MACCE** | | | |
| Random forest | 0.950 (0.948-0.952) | 0.922 (0.918-0.926) | 0.863 (0.855-0.872) |
| Gradient boosting machine | 0.994 (0.993-0.995) | 0.953 (0.951-0.956) | 0.861 (0.818-0.870) |
| Lasso Logistic Regression | 0.936 (0.934-0.937) | 0.906 (0.902-0.910) | 0.786 (0.775-0.797) |
| AdaBoost | 0.866 (0.864-0.868) | 0.866 (0.862-0.870) | 0.856 (0.848-0.865) |
| DecisionTree | 0.864 (0.861-0.867) | 0.849 (0.843-0.854) | 0.839 (0.830-0.848) |

| **Recombination (2)** | SNUBH | | AMC |
| --- | --- | --- | --- |
| AUROC (95% CI) | Train | Test | External  Validation |
| **MACCE** | | | |
| Random forest | 0.974 (0.970-0.979) | 0.894 (0.880-0.908) | 0.797 (0.764-0.800) |
| Gradient boosting machine | 0.923 (0.917-0.930) | 0.895 (0.881-0.909) | 0.815 (0.812-0.817) |
| Lasso Logistic Regression | 0.899 (0.891-0.906) | 0.889 (0.874-0.903) | 0.782 (0.779-0.785) |
| AdaBoost | 0.900 (0.893-0.907) | 0.878 (0.862-0.893) | 0.761 (0.758-0.765) |
| DecisionTree | 0.896 (0.887-0.905) | 0.809 (0.785-0.832) | 0.617 (0.613-0.622) |

| **Recombination (2)** | AMC | | SNUBH |
| --- | --- | --- | --- |
| AUROC (95% CI) | Train | Test | External  Validation |
| **MACCE** | | | |
| Random forest | 0.939 (0.937-0.941) | 0.912 (0.908-0.916) | 0.866 (0.858-0.874) |
| Gradient boosting machine | 0.992 (0.991-0.993) | 0.943 (0.940-0.946) | 0.865 (0.857-0.873) |
| Lasso Logistic Regression | 0.908 (0.906-0.911) | 0.882 (0.878-0.887) | 0.779 (0.768-0.790) |
| AdaBoost | 0.860 (0.857-0.862) | 0.858 (0.854-0.862) | 0.862 (0.854-0.870) |
| DecisionTree | 0.864 (0.861-0.867) | 0.846 (0.841-0.851) | 0.822 (0.812-0.832) |

| **Recombination (3)** | SNUBH | | AMC |
| --- | --- | --- | --- |
| AUROC (95% CI) | Train | Test | External  Validation |
| **MACCE** | | | |
| Random forest | 0.991 (0.989-0.993) | 0.837 (0.820-0.854) | 0.670 (0.666-0.674) |
| Gradient boosting machine | 0.873 (0.865-0.882) | 0.831 (0.814-0.848) | 0.694 (0.690-0.697) |
| Lasso Logistic Regression | 0.832 (0.822-0.841) | 0.824 (0.806-0.841) | 0.710 (0.707-0.713) |
| AdaBoost | 0.839 (0.830-0.848) | 0.813 (0.795-0.831) | 0.687 (0.683-0.690) |
| DecisionTree | 0.861 (0.851-0.871) | 0.707 (0.681-0.733) | 0.613 (0.609-0.616) |

| **Recombination (3)** | AMC | | SNUBH |
| --- | --- | --- | --- |
| AUROC (95% CI) | Train | Test | External  Validation |
| **MACCE** | | | |
| Random forest | 0.919 (0.916-0.921) | 0.878 (0.873-0.884) | 0.754 (0.744-0.764) |
| Gradient boosting machine | 0.991 (0.990-0.992) | 0.918 (0.914-0.922) | 0.716 (0.705-0.727) |
| Lasso Logistic Regression | 0.869 (0.866-0.871) | 0.848 (0.843-0.853) | 0.628 (0.615-0.640) |
| AdaBoost | 0.800 (0.797-0.804) | 0.800 (0.795-0.806) | 0.741 (0.731-0.752) |
| DecisionTree | 0.786 (0.782-0.789) | 0.769 (0.762-0.775) | 0.714 (0.703-0.724) |

**Table S3. MACCE standardized concept ID.**

| MACCE | CONCEPT_ID | CONCEPT_NAME | DOMAIN_ID | VOCABULARY_ID | CONCEPT_CODE |
| --- | --- | --- | --- | --- | --- |
| Stroke | 4110189 | Cerebral infarct due to thrombosis of precerebral arteries | Condition | SNOMED | 195185009 |
| Stroke | 4110190 | Cerebral infarction due to embolism of precerebral arteries | Condition | SNOMED | 195186005 |
| Stroke | 4111714 | Cerebral infarction due to cerebral venous thrombosis, non-pyogenic | Condition | SNOMED | 195230003 |
| Stroke | 4110192 | Cerebral infarction due to thrombosis of cerebral arteries | Condition | SNOMED | 195189003 |
| Stroke | 43531609 | Sequela of ischemic cerebral infarction | Condition | SNOMED | 23671000119107 |
| Stroke | 4108356 | Cerebral infarction due to embolism of cerebral arteries | Condition | SNOMED | 195190007 |
| Stroke | 4112026 | Sequelae of cerebral infarction | Condition | SNOMED | 195243003 |
| Stroke | 4045738 | Pure sensory lacunar infarction | Condition | SNOMED | 230700009 |
| Stroke | 4043731 | Infarction - precerebral | Condition | SNOMED | 230692004 |
| Stroke | 4045735 | Anterior cerebral circulation infarction | Condition | SNOMED | 230693009 |
| Stroke | 4046358 | Total anterior cerebral circulation infarction | Condition | SNOMED | 230694003 |
| Stroke | 4046359 | Partial anterior cerebral circulation infarction | Condition | SNOMED | 230695002 |
| Stroke | 4046360 | Lacunar infarction | Condition | SNOMED | 230698000 |
| Stroke | 4045737 | Pure motor lacunar infarction | Condition | SNOMED | 230699008 |
| Stroke | 4046361 | Pure sensorimotor lacunar infarction | Condition | SNOMED | 230701008 |
| Stroke | 4045740 | Lacunar ataxic hemiparesis | Condition | SNOMED | 230702001 |
| Stroke | 4046362 | Hemorrhagic cerebral infarction | Condition | SNOMED | 230706003 |
| Stroke | 4048784 | Anterior cerebral circulation hemorrhagic infarction | Condition | SNOMED | 230707007 |
| Stroke | 4043732 | Posterior cerebral circulation hemorrhagic infarction | Condition | SNOMED | 230708002 |
| Stroke | 43531592 | Sequela of thrombotic stroke | Condition | SNOMED | 91601000119109 |
| Stroke | 4194375 | Septic infarct | Observation | SNOMED | 79770005 |
| Stroke | 4319331 | Cerebellar infarction | Condition | SNOMED | 95460007 |
| Stroke | 4096252 | Impending infarction | Condition | SNOMED | 25106000 |
| Stroke | 4151368 | Edematous infarct | Observation | SNOMED | 28208008 |
| Stroke | 4141405 | Left sided cerebral infarction | Condition | SNOMED | 307766002 |
| Stroke | 4145897 | Multiple lacunar infarcts | Condition | SNOMED | 307363008 |
| Stroke | 4146185 | Right sided cerebral infarction | Condition | SNOMED | 307767006 |
| Stroke | 4077086 | Occipital cerebral infarction | Condition | SNOMED | 276219001 |
| Stroke | 46270381 | Cerebral infarction due to stenosis of precerebral artery | Condition | SNOMED | 293831000119105 |
| Stroke | 46270380 | Cerebral infarction due to vertebral artery stenosis | Condition | SNOMED | 293811000119100 |
| Stroke | 42535466 | Lacunar ataxic hemiparesis of left dominant side | Condition | SNOMED | 292861000119106 |
| Stroke | 42535465 | Lacunar ataxic hemiparesis of right dominant side | Condition | SNOMED | 292851000119109 |
| Stroke | 43530732 | Sequela of lacunar stroke | Condition | SNOMED | 33331000119103 |
| Stroke | 42535524 | Lacunar ataxic hemiparesis of right nondominant side | Condition | SNOMED | 330421000119102 |
| Stroke | 42535523 | Lacunar ataxic hemiparesis of left nondominant side | Condition | SNOMED | 330411000119109 |
| Stroke | 4267511 | Cystic infarct | Observation | SNOMED | 36560003 |
| Stroke | 46273649 | Cerebral infarction due to occlusion of basilar artery | Condition | SNOMED | 34181000119102 |
| Stroke | 44782773 | Cerebral infarction due to vertebral artery occlusion | Condition | SNOMED | 34191000119104 |
| Stroke | 765515 | Cerebral infarction due to basilar artery stenosis | Condition | SNOMED | 434991000124105 |
| Stroke | 762951 | Cerebral infarction due to anterior cerebral artery occlusion | Condition | SNOMED | 434151000124101 |
| Stroke | 4184344 | Focal infarct | Observation | SNOMED | 43618000 |
| Stroke | 763015 | Cerebral infarction due to middle cerebral artery occlusion | Condition | SNOMED | 434961000124102 |
| Stroke | 443454 | Cerebral infarction | Condition | SNOMED | 432504007 |
| Stroke | 762937 | Cerebral infarction due to cerebral venous thrombosis | Condition | SNOMED | 433961000124100 |
| Stroke | 4138327 | Acute lacunar infarction | Condition | SNOMED | 426107000 |
| Stroke | 762933 | Cerebral infarction due to cerebral artery occlusion | Condition | SNOMED | 433891000124100 |
| Stroke | 4142739 | Thalamic infarction | Condition | SNOMED | 427296003 |
| Stroke | 43530749 | Behavior disorder as sequela of cerebral infarction | Condition | SNOMED | 46421000119102 |
| Stroke | 762935 | Cerebral infarction due to internal carotid artery occlusion | Condition | SNOMED | 433931000124109 |
| Stroke | 762934 | Cerebral infarction due to posterior cerebral artery occlusion | Condition | SNOMED | 433911000124103 |
| Stroke | 4165538 | Watershed infarct | Observation | SNOMED | 47559000 |
| Stroke | 764363 | Cerebellar infarction due to occlusion of superior cerebellar artery | Condition | SNOMED | 454041000124101 |
| Stroke | 315296 | Preinfarction syndrome | Condition | SNOMED | 4557003 |
| Stroke | 4200827 | Evolving infarct | Observation | SNOMED | 52481007 |
| Stroke | 44782781 | Hemiplegia and/or hemiparesis following stroke | Condition | SNOMED | 48601000119107 |
| Stroke | 46270031 | Cerebral infarction due to occlusion of precerebral artery | Condition | SNOMED | 125081000119106 |
| Stroke | 43530683 | Cerebral infarction due to carotid artery occlusion | Condition | SNOMED | 149821000119103 |
| Stroke | 761110 | Bilateral cerebral infarction due to precererbral arterial occlusion | Condition | SNOMED | 151161000119102 |
| Stroke | 4031045 | Anterior choroidal artery syndrome | Condition | SNOMED | 14309005 |
| Stroke | 4216520 | Lacunar infarct | Observation | SNOMED | 81037000 |
| Stroke | 4251763 | Hemorrhagic infarct | Observation | SNOMED | 74310003 |
| Stroke | 765694 | Apraxia due to and following hemorrhagic cerebrovascular accident | Condition | SNOMED | 674351000119101 |
| Stroke | 42535689 | Speech and language deficit as sequela of hemorrhagic cerebrovascular accident | Condition | SNOMED | 674391000119106 |
| Stroke | 764819 | Vertigo due to and following hemorrhagic cerebrovascular accident | Condition | SNOMED | 674101000119103 |
| Stroke | 42535685 | Ataxia as sequela of hemorrhagic cerebrovascular accident | Condition | SNOMED | 674121000119107 |
| Stroke | 44783179 | Multiple lacunar infarcts | Observation | SNOMED | 698970007 |
| Stroke | 35621734 | Aphasia due to and following hemorrhagic cerebrovascular accident | Condition | SNOMED | 672551000119100 |
| Stroke | 764818 | Fluency disorder due to and following hemorrhagic cerebrovascular accident | Condition | SNOMED | 672491000119106 |
| Stroke | 42535678 | Dysarthria due to and following hemorrhagic cerebrovascular accident | Condition | SNOMED | 672511000119101 |
| Stroke | 42535680 | Dysphasia due to and following hemorrhagic cerebrovascular accident | Condition | SNOMED | 672531000119106 |
| Stroke | 45772786 | Cerebral infarction due to embolism of middle cerebral artery | Condition | SNOMED | 705128004 |
| Stroke | 45767658 | Cerebral infarction due to thrombosis of middle cerebral artery | Condition | SNOMED | 705130002 |
| Stroke | 42535682 | Cognitive deficit due to and following hemorrhagic cerebrovascular accident | Condition | SNOMED | 672571000119109 |
| Stroke | 3179950 | Right subthalamic lacunar stroke | Condition | Nebraska Lexicon | 2.16300010000041e+16 |
| Stroke | 3191217 | Chronic lacunar infarct | Observation | Nebraska Lexicon | 891011000004101 |
| Stroke | 3196370 | Acute and/or recent lacunar infarct | Observation | Nebraska Lexicon | 952381000004100 |
| Stroke | 35610084 | Cerebral infarction due to occlusion of cerebral artery | Condition | SNOMED | 1089411000000100 |
| Stroke | 1340441 | Exacerbation of preinfarction syndrome | Condition | OMOP Extension | OMOP5166096 |
| Stroke | 35610085 | Cerebral infarction due to stenosis of cerebral artery | Condition | SNOMED | 1089421000000100 |
| Stroke | 36716999 | Cerebellar stroke | Condition | SNOMED | 1.63717810001191e+16 |
| Stroke | 4111710 | Brainstem stroke syndrome | Condition | SNOMED | 195212005 |
| Stroke | 4111711 | Cerebellar stroke syndrome | Condition | SNOMED | 195213000 |
| Stroke | 381316 | Cerebrovascular accident | Condition | SNOMED | 230690007 |
| Stroke | 43531607 | Cerebral infarction due to stenosis of carotid artery | Condition | SNOMED | 99451000119105 |
| Stroke | 443790 | Multi-infarct dementia with delusions | Condition | SNOMED | 25772007 |
| Stroke | 762339 | Right anterior cerebral artery embolism with stroke | Condition | SNOMED | 329391000119100 |
| Stroke | 762340 | Left anterior cerebral artery embolism with stroke | Condition | SNOMED | 329401000119103 |
| Stroke | 762345 | Left vertebral artery thrombosis with stroke | Condition | SNOMED | 329631000119108 |
| Stroke | 4298750 | Periventricular hemorrhagic venous infarct | Condition | SNOMED | 384993003 |
| Stroke | 4159140 | Thrombotic stroke | Condition | SNOMED | 371040005 |
| Stroke | 4153352 | Embolic stroke | Condition | SNOMED | 371041009 |
| Stroke | 4310996 | Ischemic stroke | Condition | SNOMED | 422504002 |
| Stroke | 4211509 | Cardioembolic stroke | Condition | SNOMED | 413758000 |
| Stroke | 36684840 | Acute stroke | Condition | SNOMED | 457551000124104 |
| Stroke | 43531605 | Occlusion of cerebral artery with stroke | Condition | SNOMED | 9901000119100 |
| Stroke | 379778 | Multi-infarct dementia | Condition | SNOMED | 56267009 |
| Stroke | 761790 | Nonpyogenic cerebral venous thrombosis with stroke | Condition | SNOMED | 1.60021510001191e+16 |
| Stroke | 761791 | Left cerebellar artery thrombosis with stroke | Condition | SNOMED | 1.60021910001191e+16 |
| Stroke | 761793 | Left posterior cerebral artery thrombosis with stroke | Condition | SNOMED | 1.60022710001191e+16 |
| Stroke | 761794 | Right vertebral artery embolism with stroke | Condition | SNOMED | 1.60023510001191e+16 |
| Stroke | 761795 | Left vertebral artery embolism with stroke | Condition | SNOMED | 1.60023910001191e+16 |
| Stroke | 761796 | Right carotid artery embolism with stroke | Condition | SNOMED | 1.60024310001191e+16 |
| Stroke | 761797 | Left carotid artery embolism with stroke | Condition | SNOMED | 1.60024710001191e+16 |
| Stroke | 761798 | Basilar artery embolism with stroke | Condition | SNOMED | 1.60025110001191e+16 |
| Stroke | 443864 | Multi-infarct dementia with depression | Condition | SNOMED | 14070001 |
| Stroke | 43530670 | Ischemic stroke without coma | Condition | SNOMED | 140921000119102 |
| Stroke | 43530669 | Ischemic stroke with coma | Condition | SNOMED | 140911000119109 |
| Stroke | 37110237 | Cerebral ischemic stroke due to small artery occlusion | Condition | SNOMED | 724424009 |
| Stroke | 37110238 | Cerebral ischemic stroke due to intracranial large artery atherosclerosis | Condition | SNOMED | 724425005 |
| Stroke | 37110239 | Cerebral ischemic stroke due to extracranial large artery atherosclerosis | Condition | SNOMED | 724426006 |
| Stroke | 377254 | Multi-infarct dementia, uncomplicated | Condition | SNOMED | 70936005 |
| Stroke | 37110765 | Ischemic stroke without residual deficits | Condition | SNOMED | 725132001 |
| Stroke | 37110678 | Cerebral ischemic stroke due to occlusion of extracranial large artery | Condition | SNOMED | 724993002 |
| Stroke | 37110679 | Cerebral ischemic stroke due to stenosis of extracranial large artery | Condition | SNOMED | 724994008 |
| Stroke | 37395562 | Multi-infarct dementia due to atherosclerosis | Condition | SNOMED | 106021000119105 |
| Stroke | 444091 | Multi-infarct dementia with delirium | Condition | SNOMED | 10349009 |
| Myocardial infraction | 4108670 | Acute coronary insufficiency | Condition | SNOMED | 194823009 |
| Myocardial infraction | 321318 | Angina pectoris | Condition | SNOMED | 194828000 |
| Myocardial infraction | 4108679 | Rupture of cardiac wall without hemopericardium as current complication following acute myocardial infarction | Condition | SNOMED | 194865003 |
| Myocardial infraction | 4108219 | Rupture of chordae tendinae due to and following acute myocardial infarction | Condition | SNOMED | 194866002 |
| Myocardial infraction | 4108220 | Rupture of papillary muscle as current complication following acute myocardial infarction | Condition | SNOMED | 194867006 |
| Myocardial infraction | 4108680 | Thrombosis of atrium, auricular appendage, and ventricle due to and following acute myocardial infarction | Condition | SNOMED | 194868001 |
| Myocardial infraction | 4078531 | Status anginosus | Condition | SNOMED | 19057007 |
| Myocardial infraction | 4068938 | Syncope anginosa | Condition | SNOMED | 21470009 |
| Myocardial infraction | 4119942 | Stable angina | Condition | SNOMED | 233819005 |
| Myocardial infraction | 4119455 | New onset angina | Condition | SNOMED | 233821000 |
| Myocardial infraction | 4124683 | Silent myocardial ischemia | Condition | SNOMED | 233823002 |
| Myocardial infraction | 4124687 | Cardiac rupture due to and following acute myocardial infarction | Condition | SNOMED | 233847009 |
| Myocardial infraction | 4310270 | Angina, class III | Condition | SNOMED | 85284003 |
| Myocardial infraction | 42537536 | Mural thrombus of right ventricle following acute myocardial infarction | Condition | SNOMED | 736978009 |
| Myocardial infraction | 315830 | Prinzmetal angina | Condition | SNOMED | 87343002 |
| Myocardial infraction | 43020461 | Acute ST segment elevation myocardial infarction involving left main coronary artery | Condition | SNOMED | 285991000119100 |
| Myocardial infraction | 4101262 | Multiple infarcts | Observation | SNOMED | 27876002 |
| Myocardial infraction | 4147223 | Rupture of ventricle due to acute myocardial infarction | Condition | SNOMED | 30277009 |
| Myocardial infraction | 4116486 | Exercise-induced angina | Condition | SNOMED | 300995000 |
| Myocardial infraction | 4201629 | Refractory angina | Condition | SNOMED | 315025001 |
| Myocardial infraction | 4198141 | Post infarct angina | Condition | SNOMED | 314116003 |
| Myocardial infraction | 4262446 | Nocturnal angina | Condition | SNOMED | 35928006 |
| Myocardial infraction | 4261964 | Ruptured infarct | Observation | SNOMED | 35696001 |
| Myocardial infraction | 4215140 | Acute coronary syndrome | Condition | SNOMED | 394659003 |
| Myocardial infraction | 4154704 | Myocardial infarction with complication | Condition | SNOMED | 371068009 |
| Myocardial infraction | 4161973 | Progressive angina | Condition | SNOMED | 371806006 |
| Myocardial infraction | 4161974 | Recurrent angina after percutaneous transluminal coronary angioplasty | Condition | SNOMED | 371808007 |
| Myocardial infraction | 4161456 | Recurrent angina after coronary stent placement | Condition | SNOMED | 371809004 |
| Myocardial infraction | 4161457 | Recurrent angina after coronary artery bypass graft | Condition | SNOMED | 371810009 |
| Myocardial infraction | 4155008 | Recurrent angina post rotational atherectomy | Condition | SNOMED | 371811008 |
| Myocardial infraction | 4155009 | Recurrent angina after directional coronary atherectomy | Condition | SNOMED | 371812001 |
| Myocardial infraction | 4324893 | Typical angina | Condition | SNOMED | 429559004 |
| Myocardial infraction | 4186397 | Myocardial ischemia | Condition | SNOMED | 414795007 |
| Myocardial infraction | 319844 | Acute ischemic heart disease | Condition | SNOMED | 413439005 |
| Myocardial infraction | 4189939 | Acute myocardial ischemia | Condition | SNOMED | 413444003 |
| Myocardial infraction | 4184827 | Angina, class II | Condition | SNOMED | 41334000 |
| Myocardial infraction | 315286 | Chronic ischemic heart disease | Condition | SNOMED | 413838009 |
| Myocardial infraction | 4185302 | Chronic myocardial ischemia | Condition | SNOMED | 413844008 |
| Myocardial infraction | 4185932 | Ischemic heart disease | Condition | SNOMED | 414545008 |
| Myocardial infraction | 4263712 | Subendocardial ischemia | Condition | SNOMED | 46109009 |
| Myocardial infraction | 4207965 | Acute infarct | Observation | SNOMED | 55470003 |
| Myocardial infraction | 315832 | Angina decubitus | Condition | SNOMED | 59021001 |
| Myocardial infraction | 4264145 | Angina, class I | Condition | SNOMED | 61490001 |
| Myocardial infraction | 4206471 | Infarct | Observation | SNOMED | 55641003 |
| Myocardial infraction | 4209296 | Diffuse infarct | Observation | SNOMED | 56229006 |
| Myocardial infraction | 37108686 | Myocardial ischemia during surgery | Condition | SNOMED | 10971000087107 |
| Myocardial infraction | 36712983 | Angina co-occurrent and due to coronary arteriosclerosis | Condition | SNOMED | 1.59601410001191e+16 |
| Myocardial infraction | 36712984 | Angina co-occurrent and due to arteriosclerosis of coronary artery bypass graft | Condition | SNOMED | 1.59603810001191e+16 |
| Myocardial infraction | 37309713 | Unstable angina due to arteriosclerosis of autologous vein coronary artery bypass graft | Condition | SNOMED | 1.59605410001191e+16 |
| Myocardial infraction | 35615052 | Arteriosclerosis of autologous vein coronary artery bypass graft with angina | Condition | SNOMED | 1.59605810001191e+16 |
| Myocardial infraction | 35615053 | Unstable angina co-occurrent and due to arteriosclerosis of coronary artery bypass graft | Condition | SNOMED | 1.59606610001191e+16 |
| Myocardial infraction | 36712982 | Unstable angina co-occurrent and due to coronary arteriosclerosis | Condition | SNOMED | 1.59600610001191e+16 |
| Myocardial infraction | 761735 | Arteriosclerosis of autologous arterial coronary artery bypass graft with angina | Condition | SNOMED | 1.59607810001191e+16 |
| Myocardial infraction | 36712985 | Mural thrombus of left ventricle following acute myocardial infarction | Condition | SNOMED | 1.59609810001191e+16 |
| Myocardial infraction | 37209632 | Stable angina due to coronary arteriosclerosis | Condition | SNOMED | 1.67543910001191e+16 |
| Myocardial infraction | 4310118 | Transmural infarct | Observation | SNOMED | 85844000 |
| Myocardial infraction | 4231426 | Angina, class IV | Condition | SNOMED | 89323001 |
| Myocardial infraction | 43531588 | Angina associated with type 2 diabetes mellitus | Condition | SNOMED | 791000119109 |
| Myocardial infraction | 4248026 | Massive infarct | Observation | SNOMED | 72647007 |
| Myocardial infraction | 4215649 | Healed infarct | Observation | SNOMED | 71911007 |
| Myocardial infraction | 319038 | Postmyocardial infarction syndrome | Condition | SNOMED | 66189004 |
| Myocardial infraction | 4322145 | Pericarditis secondary to acute myocardial infarction | Condition | SNOMED | 71023004 |
| Myocardial infraction | 45766212 | Mitral valve regurgitation due to and following acute myocardial infarction | Condition | SNOMED | 703326006 |
| Myocardial infraction | 37017177 | Subacute ischemic heart disease | Condition | SNOMED | 713405002 |
| Myocardial infraction | 45766214 | Mitral valve regurgitation due to acute myocardial infarction without papillary muscle and chordal rupture | Condition | SNOMED | 703328007 |
| Myocardial infraction | 45771327 | Mitral valve regurgitation due to acute myocardial infarction with papillary muscle and chordal rupture | Condition | SNOMED | 703330009 |
| Myocardial infraction | 45766117 | Silent coronary vasospastic disease | Condition | SNOMED | 703214003 |
| Myocardial infraction | 4325279 | Old and recent infarct | Observation | SNOMED | 71292007 |
| Myocardial infraction | 37109910 | Ventricular aneurysm due to and following acute myocardial infarction | Condition | SNOMED | 723858002 |
| Myocardial infraction | 44783791 | Coronary microvascular dysfunction | Condition | SNOMED | 697976003 |
| Myocardial infraction | 37016755 | Resting ischemia | Condition | SNOMED | 712866001 |
| Myocardial infraction | 3195310 | Acute and/or recent infarct | Observation | Nebraska Lexicon | 952361000004106 |
| Myocardial infraction | 1340247 | Exacerbation of angina pectoris | Condition | OMOP Extension | OMOP5165902 |
| Myocardial infraction | 4011131 | Acute infarction of papillary muscle | Condition | SNOMED | 10273003 |
| Myocardial infraction | 37309626 | Myocardial infarction due to demand ischemia | Condition | SNOMED | 1.68376810001191e+16 |
| Myocardial infraction | 4329847 | Myocardial infarction | Condition | SNOMED | 22298006 |
| Myocardial infraction | 439693 | True posterior myocardial infarction | Condition | SNOMED | 194802003 |
| Myocardial infraction | 4108217 | Subsequent myocardial infarction | Condition | SNOMED | 194856005 |
| Myocardial infraction | 4108677 | Subsequent myocardial infarction of anterior wall | Condition | SNOMED | 194857001 |
| Myocardial infraction | 4108218 | Subsequent myocardial infarction of inferior wall | Condition | SNOMED | 194858006 |
| Myocardial infraction | 4108669 | Acute myocardial infarction of atrium | Condition | SNOMED | 194809007 |
| Myocardial infraction | 44782712 | Acute myocardial infarction due to right coronary artery occlusion | Condition | SNOMED | 23311000119105 |
| Myocardial infraction | 4124685 | Acute non-Q wave infarction - widespread | Condition | SNOMED | 233837006 |
| Myocardial infraction | 319039 | Acute posterior myocardial infarction | Condition | SNOMED | 233838001 |
| Myocardial infraction | 4119949 | Old anterior myocardial infarction | Condition | SNOMED | 233839009 |
| Myocardial infraction | 4121467 | Old inferior myocardial infarction | Condition | SNOMED | 233840006 |
| Myocardial infraction | 4119950 | Old lateral myocardial infarction | Condition | SNOMED | 233841005 |
| Myocardial infraction | 4119943 | Acute Q wave infarction - anteroseptal | Condition | SNOMED | 233825009 |
| Myocardial infraction | 4119456 | Acute non-Q wave infarction - anteroseptal | Condition | SNOMED | 233826005 |
| Myocardial infraction | 4119457 | Acute Q wave infarction - anterolateral | Condition | SNOMED | 233827001 |
| Myocardial infraction | 4119944 | Acute non-Q wave infarction - anterolateral | Condition | SNOMED | 233828006 |
| Myocardial infraction | 4121464 | Acute Q wave infarction - inferior | Condition | SNOMED | 233829003 |
| Myocardial infraction | 4119945 | Acute non-Q wave infarction - inferior | Condition | SNOMED | 233830008 |
| Myocardial infraction | 4121465 | Acute Q wave infarction - inferolateral | Condition | SNOMED | 233831007 |
| Myocardial infraction | 4119946 | Acute non-Q wave infarction - inferolateral | Condition | SNOMED | 233832000 |
| Myocardial infraction | 4121468 | Old posterior myocardial infarction | Condition | SNOMED | 233842003 |
| Myocardial infraction | 4124686 | Silent myocardial infarction | Condition | SNOMED | 233843008 |
| Myocardial infraction | 4124684 | Acute Q wave infarction - lateral | Condition | SNOMED | 233833005 |
| Myocardial infraction | 4121466 | Acute non-Q wave infarction - lateral | Condition | SNOMED | 233834004 |
| Myocardial infraction | 4119947 | Acute widespread myocardial infarction | Condition | SNOMED | 233835003 |
| Myocardial infraction | 4119948 | Acute Q wave infarction - widespread | Condition | SNOMED | 233836002 |
| Myocardial infraction | 312327 | Acute myocardial infarction | Condition | SNOMED | 57054005 |
| Myocardial infraction | 3661641 | Acute ST segment elevation myocardial infarction due to occlusion of proximal portion of right coronary artery | Condition | SNOMED | 868214006 |
| Myocardial infraction | 3661503 | Acute ST segment elevation myocardial infarction due to mid left anterior descending coronary artery occlusion | Condition | SNOMED | 840312002 |
| Myocardial infraction | 43020460 | Acute ST segment elevation myocardial infarction involving left anterior descending coronary artery | Condition | SNOMED | 285981000119103 |
| Myocardial infraction | 4151046 | Acute myocardial infarction of basal-lateral wall | Condition | SNOMED | 282006 |
| Myocardial infraction | 4207921 | Postoperative transmural myocardial infarction of anterior wall | Condition | SNOMED | 311792005 |
| Myocardial infraction | 4209541 | Postoperative transmural myocardial infarction of inferior wall | Condition | SNOMED | 311793000 |
| Myocardial infraction | 4206867 | Postoperative subendocardial myocardial infarction | Condition | SNOMED | 311796008 |
| Myocardial infraction | 4145721 | Acute non-Q wave infarction | Condition | SNOMED | 307140009 |
| Myocardial infraction | 4126801 | Acute Q wave myocardial infarction | Condition | SNOMED | 304914007 |
| Myocardial infraction | 4200113 | Non-Q wave myocardial infarction | Condition | SNOMED | 314207007 |
| Myocardial infraction | 4138833 | Past myocardial infarction diagnosed on ECG AND/OR other special investigation, but currently presenting no symptoms | Condition | SNOMED | 32574007 |
| Myocardial infraction | 4215259 | First myocardial infarction | Condition | SNOMED | 394710008 |
| Myocardial infraction | 765132 | Subendocardial myocardial infarction | Condition | SNOMED | 380001000004106 |
| Myocardial infraction | 4296653 | Acute ST segment elevation myocardial infarction | Condition | SNOMED | 401303003 |
| Myocardial infraction | 4270024 | Acute non-ST segment elevation myocardial infarction | Condition | SNOMED | 401314000 |
| Myocardial infraction | 4323202 | Mixed myocardial ischemia and infarction | Condition | SNOMED | 428196007 |
| Myocardial infraction | 4173632 | Microinfarct of heart | Condition | SNOMED | 42531007 |
| Myocardial infraction | 4178129 | Acute anteroapical myocardial infarction | Condition | SNOMED | 52035003 |
| Myocardial infraction | 4267568 | Acute anteroseptal myocardial infarction | Condition | SNOMED | 62695002 |
| Myocardial infraction | 436706 | Acute myocardial infarction of lateral wall | Condition | SNOMED | 58612006 |
| Myocardial infraction | 4170094 | Myocardial infarction in recovery phase | Condition | SNOMED | 418044006 |
| Myocardial infraction | 434376 | Acute myocardial infarction of anterior wall | Condition | SNOMED | 54329005 |
| Myocardial infraction | 4243372 | Acute myocardial infarction of apical-lateral wall | Condition | SNOMED | 59063002 |
| Myocardial infraction | 35610091 | Acute nontransmural myocardial infarction | Condition | SNOMED | 1089451000000100 |
| Myocardial infraction | 35610093 | Acute transmural myocardial infarction | Condition | SNOMED | 1089471000000100 |
| Myocardial infraction | 35611570 | Acute ST segment elevation myocardial infarction of inferolateral wall | Condition | SNOMED | 1.22381110001191e+16 |
| Myocardial infraction | 35611571 | Acute ST segment elevation myocardial infarction of inferoposterior wall | Condition | SNOMED | 1.22381510001191e+16 |
| Myocardial infraction | 761737 | Acute ST segment elevation myocardial infarction due to occlusion of circumflex coronary artery | Condition | SNOMED | 1.59631810001191e+16 |
| Myocardial infraction | 4030582 | Postoperative myocardial infarction | Condition | SNOMED | 129574000 |
| Myocardial infraction | 761736 | Acute ST segment elevation myocardial infarction of anteroapical wall | Condition | SNOMED | 1.59625410001191e+16 |
| Myocardial infraction | 4051874 | Acute myocardial infarction of posterolateral wall | Condition | SNOMED | 15990001 |
| Myocardial infraction | 46270158 | Acute ST segment elevation myocardial infarction of posterolateral wall | Condition | SNOMED | 1.57128410001191e+16 |
| Myocardial infraction | 46270163 | Acute ST segment elevation myocardial infarction due to right coronary artery occlusion | Condition | SNOMED | 1.57131210001191e+16 |
| Myocardial infraction | 46270164 | Acute ST segment elevation myocardial infarction of septum | Condition | SNOMED | 1.57131610001191e+16 |
| Myocardial infraction | 46273495 | Acute ST segment elevation myocardial infarction of posterobasal wall | Condition | SNOMED | 1.57132010001191e+16 |
| Myocardial infraction | 46270159 | Acute ST segment elevation myocardial infarction of anterolateral wall | Condition | SNOMED | 1.57128810001191e+16 |
| Myocardial infraction | 46274044 | Acute ST segment elevation myocardial infarction of lateral wall | Condition | SNOMED | 1.57129210001191e+16 |
| Myocardial infraction | 46270160 | Acute ST segment elevation myocardial infarction of anteroseptal wall | Condition | SNOMED | 1.57129610001191e+16 |
| Myocardial infraction | 46270161 | Acute ST segment elevation myocardial infarction of posterior wall | Condition | SNOMED | 1.57130410001191e+16 |
| Myocardial infraction | 46270162 | Acute ST segment elevation myocardial infarction due to left coronary artery occlusion | Condition | SNOMED | 1.57130810001191e+16 |
| Myocardial infraction | 44782769 | Acute myocardial infarction due to left coronary artery occlusion | Condition | SNOMED | 17531000119105 |
| Myocardial infraction | 314666 | Old myocardial infarction | Condition | SNOMED | 1755008 |
| Myocardial infraction | 3661502 | Acute ST segment elevation myocardial infarction due to proximal left anterior descending coronary artery occlusion | Condition | SNOMED | 840309000 |
| Myocardial infraction | 3661504 | Acute ST segment elevation myocardial infarction due to distal left anterior descending coronary artery occlusion | Condition | SNOMED | 840316004 |
| Myocardial infraction | 3661642 | Acute ST segment elevation myocardial infarction due to occlusion of distal portion of right coronary artery | Condition | SNOMED | 868217004 |
| Myocardial infraction | 3661643 | Acute ST segment elevation myocardial infarction due to occlusion of mid portion of right coronary artery | Condition | SNOMED | 868220007 |
| Myocardial infraction | 3661644 | Acute ST segment elevation myocardial infarction due to occlusion of marginal branch of right coronary artery | Condition | SNOMED | 868224003 |
| Myocardial infraction | 3661645 | Acute ST segment elevation myocardial infarction due to occlusion of posterior descending branch of right coronary artery | Condition | SNOMED | 868225002 |
| Myocardial infraction | 3661646 | Acute ST segment elevation myocardial infarction due to occlusion of posterior lateral branch of right coronary artery | Condition | SNOMED | 868226001 |
| Myocardial infraction | 3655133 | Acute ST segment elevation myocardial infarction due to occlusion of intermediate artery | Condition | SNOMED | 846683001 |
| Myocardial infraction | 3661547 | Acute ST segment elevation myocardial infarction due to occlusion of diagonal branch of anterior descending branch of left coronary artery | Condition | SNOMED | 846668006 |
| Myocardial infraction | 3661520 | Acute ST segment elevation myocardial infarction due to occlusion of anterior descending branch of left coronary artery | Condition | SNOMED | 840609007 |
| Myocardial infraction | 441579 | Acute myocardial infarction of inferoposterior wall | Condition | SNOMED | 76593002 |
| Myocardial infraction | 3661524 | Acute ST segment elevation myocardial infarction due to occlusion of septal branch of anterior descending branch of left coronary artery | Condition | SNOMED | 840680009 |
| Myocardial infraction | 438170 | Acute myocardial infarction of inferior wall | Condition | SNOMED | 73795002 |
| Myocardial infraction | 3654465 | Acute myocardial infarction of right ventricle | Condition | SNOMED | 836293000 |
| Myocardial infraction | 4303359 | Acute myocardial infarction of septum | Condition | SNOMED | 79009004 |
| Myocardial infraction | 3654466 | Acute myocardial infarction of apex of heart | Condition | SNOMED | 836294006 |
| Myocardial infraction | 3654467 | Acute myocardial infarction of inferolateral wall with posterior extension | Condition | SNOMED | 836295007 |
| Myocardial infraction | 4324413 | Acute myocardial infarction of posterobasal wall | Condition | SNOMED | 70998009 |
| Myocardial infraction | 45771322 | Acute myocardial infarction of inferior wall involving right ventricle | Condition | SNOMED | 703251009 |
| Myocardial infraction | 45766150 | Acute myocardial infarction of anterior wall involving right ventricle | Condition | SNOMED | 703252002 |
| Myocardial infraction | 45766151 | Acute ST segment elevation myocardial infarction of inferior wall involving right ventricle | Condition | SNOMED | 703253007 |
| Myocardial infraction | 4275436 | Acute myocardial infarction of high lateral wall | Condition | SNOMED | 64627002 |
| Myocardial infraction | 45766241 | Subsequent non-ST segment elevation myocardial infarction | Condition | SNOMED | 703360004 |
| Myocardial infraction | 45766075 | Acute anterior ST segment elevation myocardial infarction | Condition | SNOMED | 703164000 |
| Myocardial infraction | 45766076 | Acute ST segment elevation myocardial infarction of anterior wall involving right ventricle | Condition | SNOMED | 703165004 |
| Myocardial infraction | 45773170 | Subsequent ST segment elevation myocardial infarction of inferior wall | Condition | SNOMED | 703209002 |
| Myocardial infraction | 45766113 | Subsequent ST segment elevation myocardial infarction of anterior wall | Condition | SNOMED | 703210007 |
| Myocardial infraction | 45766114 | Subsequent ST segment elevation myocardial infarction | Condition | SNOMED | 703211006 |
| Myocardial infraction | 45766115 | Acute myocardial infarction during procedure | Condition | SNOMED | 703212004 |
| Myocardial infraction | 45766116 | Acute ST segment elevation myocardial infarction of inferior wall | Condition | SNOMED | 703213009 |
| Myocardial infraction | 438438 | Acute myocardial infarction of anterolateral wall | Condition | SNOMED | 70211005 |
| Myocardial infraction | 444406 | Acute subendocardial infarction | Condition | SNOMED | 70422006 |
| Myocardial infraction | 438447 | Acute myocardial infarction of inferolateral wall | Condition | SNOMED | 65547006 |
| Myocardial infraction | 3189643 | Anterior subendocardial myocardial infarction | Condition | Nebraska Lexicon | 2.97800010000041e+16 |
| Myocardial infraction | 35610087 | Postoperative nontransmural myocardial infarction | Condition | SNOMED | 1089431000000100 |
| Myocardial infraction | 35610089 | Postoperative transmural myocardial infarction | Condition | SNOMED | 1089441000000100 |
| Heart failure | 37309625 | Acute on chronic right-sided congestive heart failure | Condition | SNOMED | 1.68389510001191e+16 |
| Heart failure | 37309625 | Acute on chronic right-sided congestive heart failure | Condition | SNOMED | 1.68389510001191e+16 |
| Heart failure | 4071869 | Congenital cardiac failure | Condition | SNOMED | 206586007 |
| Heart failure | 4071869 | Congenital cardiac failure | Condition | SNOMED | 206586007 |
| Heart failure | 439696 | Hypertensive heart and renal disease with (congestive) heart failure | Condition | SNOMED | 194779001 |
| Heart failure | 439696 | Hypertensive heart and renal disease with (congestive) heart failure | Condition | SNOMED | 194779001 |
| Heart failure | 4111554 | Decompensated cardiac failure | Condition | SNOMED | 195111005 |
| Heart failure | 4108245 | Acute left ventricular failure | Condition | SNOMED | 195114002 |
| Heart failure | 4108245 | Acute left ventricular failure | Condition | SNOMED | 195114002 |
| Heart failure | 4111554 | Decompensated cardiac failure | Condition | SNOMED | 195111005 |
| Heart failure | 4108244 | Compensated cardiac failure | Condition | SNOMED | 195112003 |
| Heart failure | 4108244 | Compensated cardiac failure | Condition | SNOMED | 195112003 |
| Heart failure | 439698 | Benign hypertensive heart disease with congestive cardiac failure | Condition | SNOMED | 194767001 |
| Heart failure | 439694 | Hypertensive heart and renal disease with both (congestive) heart failure and renal failure | Condition | SNOMED | 194781004 |
| Heart failure | 439694 | Hypertensive heart and renal disease with both (congestive) heart failure and renal failure | Condition | SNOMED | 194781004 |
| Heart failure | 439698 | Benign hypertensive heart disease with congestive cardiac failure | Condition | SNOMED | 194767001 |
| Heart failure | 44782713 | Congestive heart failure with right heart failure | Condition | SNOMED | 23341000119109 |
| Heart failure | 44782713 | Congestive heart failure with right heart failure | Condition | SNOMED | 23341000119109 |
| Heart failure | 4124705 | Heart failure as a complication of care | Condition | SNOMED | 233924009 |
| Heart failure | 4124705 | Heart failure as a complication of care | Condition | SNOMED | 233924009 |
| Heart failure | 43020657 | Heart failure due to end stage congenital heart disease | Condition | SNOMED | 471880001 |
| Heart failure | 42598803 | Spontaneous cardiomyopathy | Condition | SNOMED Veterinary | 339881000009101 |
| Heart failure | 42598803 | Spontaneous cardiomyopathy | Condition | SNOMED Veterinary | 339881000009101 |
| Heart failure | 3656094 | Low output heart failure due to and following Fontan operation | Condition | SNOMED | 871617000 |
| Heart failure | 4242669 | Biventricular congestive heart failure | Condition | SNOMED | 92506005 |
| Heart failure | 4242669 | Biventricular congestive heart failure | Condition | SNOMED | 92506005 |
| Heart failure | 3656094 | Low output heart failure due to and following Fontan operation | Condition | SNOMED | 871617000 |
| Heart failure | 439846 | Left heart failure | Condition | SNOMED | 85232009 |
| Heart failure | 439846 | Left heart failure | Condition | SNOMED | 85232009 |
| Heart failure | 764877 | Acute heart failure co-occurrent with normal ejection fraction | Condition | SNOMED | 7421000175106 |
| Heart failure | 764877 | Acute heart failure co-occurrent with normal ejection fraction | Condition | SNOMED | 7421000175106 |
| Heart failure | 4229440 | Chronic congestive heart failure | Condition | SNOMED | 88805009 |
| Heart failure | 4229440 | Chronic congestive heart failure | Condition | SNOMED | 88805009 |
| Heart failure | 764871 | Reduced ejection fraction co-occurrent and due to acute on chronic heart failure | Condition | SNOMED | 7371000175103 |
| Heart failure | 764871 | Reduced ejection fraction co-occurrent and due to acute on chronic heart failure | Condition | SNOMED | 7371000175103 |
| Heart failure | 43022068 | Exacerbation of congestive heart failure | Condition | SNOMED | 96311000119109 |
| Heart failure | 43022068 | Exacerbation of congestive heart failure | Condition | SNOMED | 96311000119109 |
| Heart failure | 762002 | Congestive heart failure as post-operative complication of cardiac surgery | Condition | SNOMED | 285211000119102 |
| Heart failure | 762002 | Congestive heart failure as post-operative complication of cardiac surgery | Condition | SNOMED | 285211000119102 |
| Heart failure | 762003 | Congestive heart failure as post-operative complication of non-cardiac surgery | Condition | SNOMED | 285221000119109 |
| Heart failure | 762003 | Congestive heart failure as post-operative complication of non-cardiac surgery | Condition | SNOMED | 285221000119109 |
| Heart failure | 4103448 | Low output heart failure | Condition | SNOMED | 25544003 |
| Heart failure | 4103448 | Low output heart failure | Condition | SNOMED | 25544003 |
| Heart failure | 4079695 | Sepsis-associated left ventricular failure | Condition | SNOMED | 277638005 |
| Heart failure | 4079695 | Sepsis-associated left ventricular failure | Condition | SNOMED | 277638005 |
| Heart failure | 4079296 | Sepsis-associated right ventricular failure | Condition | SNOMED | 277639002 |
| Heart failure | 4079296 | Sepsis-associated right ventricular failure | Condition | SNOMED | 277639002 |
| Heart failure | 4199500 | Refractory heart failure | Condition | SNOMED | 314206003 |
| Heart failure | 4199500 | Refractory heart failure | Condition | SNOMED | 314206003 |
| Heart failure | 4273632 | Right ventricular failure | Condition | SNOMED | 367363000 |
| Heart failure | 4273632 | Right ventricular failure | Condition | SNOMED | 367363000 |
| Heart failure | 314369 | Malignant hypertensive heart disease without congestive heart failure | Condition | SNOMED | 36315003 |
| Heart failure | 4267800 | Acute left-sided heart failure | Condition | SNOMED | 364006 |
| Heart failure | 4267800 | Acute left-sided heart failure | Condition | SNOMED | 364006 |
| Heart failure | 4233424 | Acute right-sided heart failure | Condition | SNOMED | 359617009 |
| Heart failure | 4233424 | Acute right-sided heart failure | Condition | SNOMED | 359617009 |
| Heart failure | 4185565 | Low cardiac output syndrome | Condition | SNOMED | 44088000 |
| Heart failure | 4185565 | Low cardiac output syndrome | Condition | SNOMED | 44088000 |
| Heart failure | 40482857 | Cardiorenal syndrome | Condition | SNOMED | 445236007 |
| Heart failure | 40479192 | Chronic systolic heart failure | Condition | SNOMED | 441481004 |
| Heart failure | 40479192 | Chronic systolic heart failure | Condition | SNOMED | 441481004 |
| Heart failure | 40481042 | Acute diastolic heart failure | Condition | SNOMED | 443343001 |
| Heart failure | 40481042 | Acute diastolic heart failure | Condition | SNOMED | 443343001 |
| Heart failure | 40481043 | Acute on chronic diastolic heart failure | Condition | SNOMED | 443344007 |
| Heart failure | 40481043 | Acute on chronic diastolic heart failure | Condition | SNOMED | 443344007 |
| Heart failure | 40479576 | Chronic diastolic heart failure | Condition | SNOMED | 441530006 |
| Heart failure | 40479576 | Chronic diastolic heart failure | Condition | SNOMED | 441530006 |
| Heart failure | 4195785 | Right heart failure secondary to left heart failure | Condition | SNOMED | 44313006 |
| Heart failure | 4195785 | Right heart failure secondary to left heart failure | Condition | SNOMED | 44313006 |
| Heart failure | 40480602 | Acute on chronic systolic heart failure | Condition | SNOMED | 443253003 |
| Heart failure | 40480602 | Acute on chronic systolic heart failure | Condition | SNOMED | 443253003 |
| Heart failure | 40480603 | Acute systolic heart failure | Condition | SNOMED | 443254009 |
| Heart failure | 40480603 | Acute systolic heart failure | Condition | SNOMED | 443254009 |
| Heart failure | 40482857 | Cardiorenal syndrome | Condition | SNOMED | 445236007 |
| Heart failure | 40486933 | Heart failure with normal ejection fraction | Condition | SNOMED | 446221000 |
| Heart failure | 40486933 | Heart failure with normal ejection fraction | Condition | SNOMED | 446221000 |
| Heart failure | 4184497 | Rheumatic left ventricular failure | Condition | SNOMED | 43736008 |
| Heart failure | 4184497 | Rheumatic left ventricular failure | Condition | SNOMED | 43736008 |
| Heart failure | 4259490 | Cardiorespiratory failure | Condition | SNOMED | 410431009 |
| Heart failure | 4259490 | Cardiorespiratory failure | Condition | SNOMED | 410431009 |
| Heart failure | 4142561 | Congestive heart failure due to valvular disease | Condition | SNOMED | 426611007 |
| Heart failure | 4142561 | Congestive heart failure due to valvular disease | Condition | SNOMED | 426611007 |
| Heart failure | 4139864 | Congestive heart failure due to left ventricular systolic dysfunction | Condition | SNOMED | 426263006 |
| Heart failure | 4139864 | Congestive heart failure due to left ventricular systolic dysfunction | Condition | SNOMED | 426263006 |
| Heart failure | 4311437 | Decompensated chronic heart failure | Condition | SNOMED | 424404003 |
| Heart failure | 4311437 | Decompensated chronic heart failure | Condition | SNOMED | 424404003 |
| Heart failure | 4138307 | Right heart failure due to pulmonary hypertension | Condition | SNOMED | 426012001 |
| Heart failure | 4138307 | Right heart failure due to pulmonary hypertension | Condition | SNOMED | 426012001 |
| Heart failure | 444031 | Chronic heart failure | Condition | SNOMED | 48447003 |
| Heart failure | 444031 | Chronic heart failure | Condition | SNOMED | 48447003 |
| Heart failure | 4205558 | Cardiac failure after obstetrical surgery AND/OR other procedure including delivery | Condition | SNOMED | 55565007 |
| Heart failure | 4205558 | Cardiac failure after obstetrical surgery AND/OR other procedure including delivery | Condition | SNOMED | 55565007 |
| Heart failure | 443580 | Systolic heart failure | Condition | SNOMED | 417996009 |
| Heart failure | 443580 | Systolic heart failure | Condition | SNOMED | 417996009 |
| Heart failure | 443587 | Diastolic heart failure | Condition | SNOMED | 418304008 |
| Heart failure | 443587 | Diastolic heart failure | Condition | SNOMED | 418304008 |
| Heart failure | 444101 | Hypertensive heart failure | Condition | SNOMED | 46113002 |
| Heart failure | 43021735 | Fetal heart failure | Condition | SNOMED | 462172006 |
| Heart failure | 43021735 | Fetal heart failure | Condition | SNOMED | 462172006 |
| Heart failure | 43022054 | Fetal heart failure with myocardial hypertrophy | Condition | SNOMED | 462174007 |
| Heart failure | 43022054 | Fetal heart failure with myocardial hypertrophy | Condition | SNOMED | 462174007 |
| Heart failure | 43021736 | Fetal heart failure with redistribution of cardiac output | Condition | SNOMED | 462175008 |
| Heart failure | 43021736 | Fetal heart failure with redistribution of cardiac output | Condition | SNOMED | 462175008 |
| Heart failure | 444101 | Hypertensive heart failure | Condition | SNOMED | 46113002 |
| Heart failure | 319835 | Congestive heart failure | Condition | SNOMED | 42343007 |
| Heart failure | 319835 | Congestive heart failure | Condition | SNOMED | 42343007 |
| Heart failure | 43530961 | Induced termination of pregnancy complicated by cardiac failure | Condition | SNOMED | 609507007 |
| Heart failure | 442310 | Acute heart failure | Condition | SNOMED | 56675007 |
| Heart failure | 442310 | Acute heart failure | Condition | SNOMED | 56675007 |
| Heart failure | 43530961 | Induced termination of pregnancy complicated by cardiac failure | Condition | SNOMED | 609507007 |
| Heart failure | 319034 | Hypertensive heart disease without congestive heart failure | Condition | SNOMED | 60899001 |
| Heart failure | 4206009 | Chronic left-sided congestive heart failure | Condition | SNOMED | 5375005 |
| Heart failure | 4206009 | Chronic left-sided congestive heart failure | Condition | SNOMED | 5375005 |
| Heart failure | 314378 | Hypertensive heart disease with congestive heart failure | Condition | SNOMED | 5148006 |
| Heart failure | 314378 | Hypertensive heart disease with congestive heart failure | Condition | SNOMED | 5148006 |
| Heart failure | 43020657 | Heart failure due to end stage congenital heart disease | Condition | SNOMED | 471880001 |
| Heart failure | 4009047 | Chronic left-sided heart failure | Condition | SNOMED | 111283005 |
| Heart failure | 4009047 | Chronic left-sided heart failure | Condition | SNOMED | 111283005 |
| Heart failure | 43021840 | Systolic heart failure stage D | Condition | SNOMED | 120851000119104 |
| Heart failure | 43530642 | Systolic heart failure stage B | Condition | SNOMED | 120871000119108 |
| Heart failure | 43530642 | Systolic heart failure stage B | Condition | SNOMED | 120871000119108 |
| Heart failure | 43021840 | Systolic heart failure stage D | Condition | SNOMED | 120851000119104 |
| Heart failure | 43020421 | Systolic heart failure stage C | Condition | SNOMED | 120861000119102 |
| Heart failure | 43020421 | Systolic heart failure stage C | Condition | SNOMED | 120861000119102 |
| Heart failure | 43021842 | Diastolic heart failure stage C | Condition | SNOMED | 120891000119109 |
| Heart failure | 43021841 | Diastolic heart failure stage D | Condition | SNOMED | 120881000119106 |
| Heart failure | 43021841 | Diastolic heart failure stage D | Condition | SNOMED | 120881000119106 |
| Heart failure | 43021842 | Diastolic heart failure stage C | Condition | SNOMED | 120891000119109 |
| Heart failure | 43530643 | Diastolic heart failure stage B | Condition | SNOMED | 120901000119108 |
| Heart failure | 43530643 | Diastolic heart failure stage B | Condition | SNOMED | 120901000119108 |
| Heart failure | 44782728 | Hypertensive heart AND chronic kidney disease with congestive heart failure | Condition | SNOMED | 15781000119107 |
| Heart failure | 44782728 | Hypertensive heart AND chronic kidney disease with congestive heart failure | Condition | SNOMED | 15781000119107 |
| Heart failure | 44782719 | Chronic combined systolic and diastolic heart failure | Condition | SNOMED | 153941000119100 |
| Heart failure | 44782718 | Acute combined systolic and diastolic heart failure | Condition | SNOMED | 153931000119109 |
| Heart failure | 44782718 | Acute combined systolic and diastolic heart failure | Condition | SNOMED | 153931000119109 |
| Heart failure | 44782719 | Chronic combined systolic and diastolic heart failure | Condition | SNOMED | 153941000119100 |
| Heart failure | 44782733 | Acute on chronic combined systolic and diastolic heart failure | Condition | SNOMED | 153951000119103 |
| Heart failure | 44782733 | Acute on chronic combined systolic and diastolic heart failure | Condition | SNOMED | 153951000119103 |
| Heart failure | 36717359 | Systolic heart failure stage B due to ischemic cardiomyopathy | Condition | SNOMED | 1.56296410001191e+16 |
| Heart failure | 36717359 | Systolic heart failure stage B due to ischemic cardiomyopathy | Condition | SNOMED | 1.56296410001191e+16 |
| Heart failure | 36712929 | Systolic heart failure stage C due to ischemic cardiomyopathy | Condition | SNOMED | 1.56297410001191e+16 |
| Heart failure | 36712929 | Systolic heart failure stage C due to ischemic cardiomyopathy | Condition | SNOMED | 1.56297410001191e+16 |
| Heart failure | 36712927 | Congestive heart failure stage C due to ischemic cardiomyopathy | Condition | SNOMED | 1.56295410001191e+16 |
| Heart failure | 36712927 | Congestive heart failure stage C due to ischemic cardiomyopathy | Condition | SNOMED | 1.56295410001191e+16 |
| Heart failure | 36712928 | Congestive heart failure stage B due to ischemic cardiomyopathy | Condition | SNOMED | 1.56295910001191e+16 |
| Heart failure | 36712928 | Congestive heart failure stage B due to ischemic cardiomyopathy | Condition | SNOMED | 1.56295910001191e+16 |
| Heart failure | 764873 | Reduced ejection fraction co-occurrent and due to acute heart failure | Condition | SNOMED | 7391000175102 |
| Heart failure | 4327205 | Acute left-sided congestive heart failure | Condition | SNOMED | 74960003 |
| Heart failure | 4327205 | Acute left-sided congestive heart failure | Condition | SNOMED | 74960003 |
| Heart failure | 764873 | Reduced ejection fraction co-occurrent and due to acute heart failure | Condition | SNOMED | 7391000175102 |
| Heart failure | 313502 | Benign hypertensive heart disease without congestive heart failure | Condition | SNOMED | 77970009 |
| Heart failure | 316139 | Heart failure | Condition | SNOMED | 84114007 |
| Heart failure | 316139 | Heart failure | Condition | SNOMED | 84114007 |
| Heart failure | 764876 | Chronic heart failure co-occurrent with normal ejection fraction | Condition | SNOMED | 7411000175102 |
| Heart failure | 764872 | Reduced ejection fraction co-occurrent and due to chronic heart failure | Condition | SNOMED | 7381000175100 |
| Heart failure | 764872 | Reduced ejection fraction co-occurrent and due to chronic heart failure | Condition | SNOMED | 7381000175100 |
| Heart failure | 764876 | Chronic heart failure co-occurrent with normal ejection fraction | Condition | SNOMED | 7411000175102 |
| Heart failure | 4219755 | Ischemic contracture of left ventricle syndrome | Condition | SNOMED | 82522008 |
| Heart failure | 315295 | Congestive rheumatic heart failure | Condition | SNOMED | 82523003 |
| Heart failure | 315295 | Congestive rheumatic heart failure | Condition | SNOMED | 82523003 |
| Heart failure | 316994 | Malignant hypertensive heart disease with congestive heart failure | Condition | SNOMED | 83105008 |
| Heart failure | 316994 | Malignant hypertensive heart disease with congestive heart failure | Condition | SNOMED | 83105008 |
| Heart failure | 37311948 | Heart failure with mid range ejection fraction | Condition | SNOMED | 788950000 |
| Heart failure | 37311948 | Heart failure with mid range ejection fraction | Condition | SNOMED | 788950000 |
| Heart failure | 45766164 | Heart failure with reduced ejection fraction | Condition | SNOMED | 703272007 |
| Heart failure | 45766164 | Heart failure with reduced ejection fraction | Condition | SNOMED | 703272007 |
| Heart failure | 45766165 | Heart failure with reduced ejection fraction due to coronary artery disease | Condition | SNOMED | 703273002 |
| Heart failure | 45766165 | Heart failure with reduced ejection fraction due to coronary artery disease | Condition | SNOMED | 703273002 |
| Heart failure | 45766166 | Heart failure with reduced ejection fraction due to myocarditis | Condition | SNOMED | 703274008 |
| Heart failure | 45766166 | Heart failure with reduced ejection fraction due to myocarditis | Condition | SNOMED | 703274008 |
| Heart failure | 45766167 | Heart failure with reduced ejection fraction due to cardiomyopathy | Condition | SNOMED | 703275009 |
| Heart failure | 45766167 | Heart failure with reduced ejection fraction due to cardiomyopathy | Condition | SNOMED | 703275009 |
| Heart failure | 45773075 | Heart failure with reduced ejection fraction due to heart valve disease | Condition | SNOMED | 703276005 |
| Heart failure | 45773075 | Heart failure with reduced ejection fraction due to heart valve disease | Condition | SNOMED | 703276005 |
| Heart failure | 44784442 | Symptomatic congestive heart failure | Condition | SNOMED | 698594003 |
| Heart failure | 44784442 | Symptomatic congestive heart failure | Condition | SNOMED | 698594003 |
| Heart failure | 44782655 | Acute exacerbation of chronic congestive heart failure | Condition | SNOMED | 698296002 |
| Heart failure | 44782655 | Acute exacerbation of chronic congestive heart failure | Condition | SNOMED | 698296002 |
| Heart failure | 764874 | Acute on chronic heart failure co-occurrent with normal ejection fraction | Condition | SNOMED | 7401000175100 |
| Heart failure | 764874 | Acute on chronic heart failure co-occurrent with normal ejection fraction | Condition | SNOMED | 7401000175100 |
| Heart failure | 4215446 | Acute right-sided congestive heart failure | Condition | SNOMED | 80479009 |
| Heart failure | 4215446 | Acute right-sided congestive heart failure | Condition | SNOMED | 80479009 |
| Heart failure | 4215802 | Cardiac asthma | Condition | SNOMED | 71892000 |
| Heart failure | 4215802 | Cardiac asthma | Condition | SNOMED | 71892000 |
| Heart failure | 36713488 | Congestive heart failure stage B | Condition | SNOMED | 717840005 |
| Heart failure | 36713488 | Congestive heart failure stage B | Condition | SNOMED | 717840005 |
| Heart failure | 43021826 | Congestive heart failure stage C | Condition | SNOMED | 67441000119101 |
| Heart failure | 43021826 | Congestive heart failure stage C | Condition | SNOMED | 67441000119101 |
| Heart failure | 43021825 | Congestive heart failure stage D | Condition | SNOMED | 67431000119105 |
| Heart failure | 43021825 | Congestive heart failure stage D | Condition | SNOMED | 67431000119105 |
| Heart failure | 45766964 | Fetal heart failure due to extracardiac disease | Condition | SNOMED | 704242009 |
| Heart failure | 4284562 | Chronic right-sided congestive heart failure | Condition | SNOMED | 66989003 |
| Heart failure | 4284562 | Chronic right-sided congestive heart failure | Condition | SNOMED | 66989003 |
| Heart failure | 36716182 | Acute kidney injury due to circulatory failure | Condition | SNOMED | 722095005 |
| Heart failure | 36716182 | Acute kidney injury due to circulatory failure | Condition | SNOMED | 722095005 |
| Heart failure | 44784345 | Congestive heart failure as early postoperative complication | Condition | SNOMED | 72481000119103 |
| Heart failure | 44784345 | Congestive heart failure as early postoperative complication | Condition | SNOMED | 72481000119103 |
| Heart failure | 3184320 | Heart failure exacerbated by Sotalol | Condition | Nebraska Lexicon | 1.26100010000041e+16 |
| Heart failure | 3184320 | Heart failure exacerbated by Sotalol | Condition | Nebraska Lexicon | 1.26100010000041e+16 |
| Heart failure | 1340348 | Exacerbation of heart failure | Condition | OMOP Extension | OMOP5166003 |
| Heart failure | 1340281 | Exacerbation of chronic heart failure | Condition | OMOP Extension | OMOP5165936 |
| Heart failure | 1340281 | Exacerbation of chronic heart failure | Condition | OMOP Extension | OMOP5165936 |
| Heart failure | 1340289 | Exacerbation of congestive heart failure | Condition | OMOP Extension | OMOP5165944 |
| Heart failure | 1340289 | Exacerbation of congestive heart failure | Condition | OMOP Extension | OMOP5165944 |
| Heart failure | 1340348 | Exacerbation of heart failure | Condition | OMOP Extension | OMOP5166003 |
| Heart failure | 4014159 | Chronic right-sided heart failure | Condition | SNOMED | 10335000 |
| Heart failure | 4014159 | Chronic right-sided heart failure | Condition | SNOMED | 10335000 |
| Heart failure | 44782428 | Congestive heart failure due to cardiomyopathy | Condition | SNOMED | 101281000119107 |
| Heart failure | 44782428 | Congestive heart failure due to cardiomyopathy | Condition | SNOMED | 101281000119107 |
| Heart failure | 4004279 | High output heart failure | Condition | SNOMED | 10091002 |
| Heart failure | 4004279 | High output heart failure | Condition | SNOMED | 10091002 |
| Heart failure | 4023479 | Acute congestive heart failure | Condition | SNOMED | 10633002 |
| Heart failure | 4023479 | Acute congestive heart failure | Condition | SNOMED | 10633002 |
| Death | 4317150 | cardiac death | Condition | SNOMED | 95281009 |
| Cardiac arrest/shock | 321042 | Cardiac arrest | Condition | SNOMED | 410429000 |

**
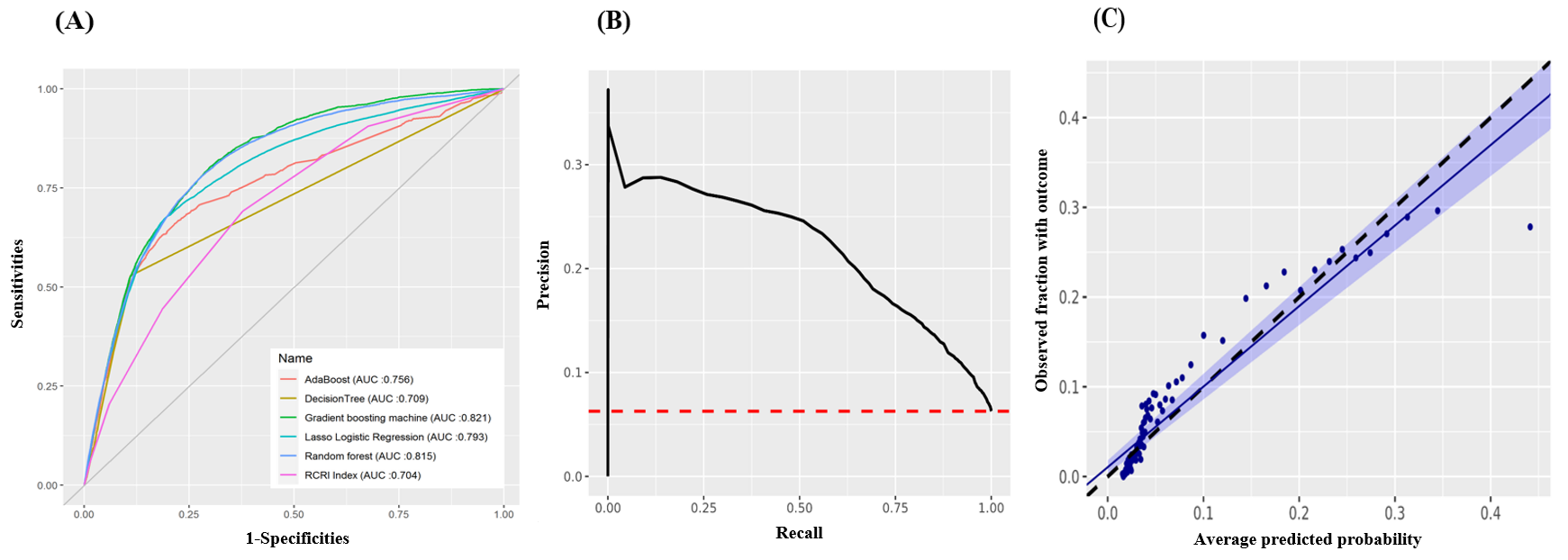
**

**Figure S1. Recombination prediction model (1) based on validation data**

(A) AUROC for predicting MACCE, demonstrating the model's ability to distinguish between patients with and without MACCE

(B) AUPRC for predicting MACCE, illustrating the precision-recall trade-off of the model (Gradient boosting machine).

(C) Calibration plot for MACCE, assessing the agreement between predicted probabilities and observed outcomes (Gradient boosting machine).


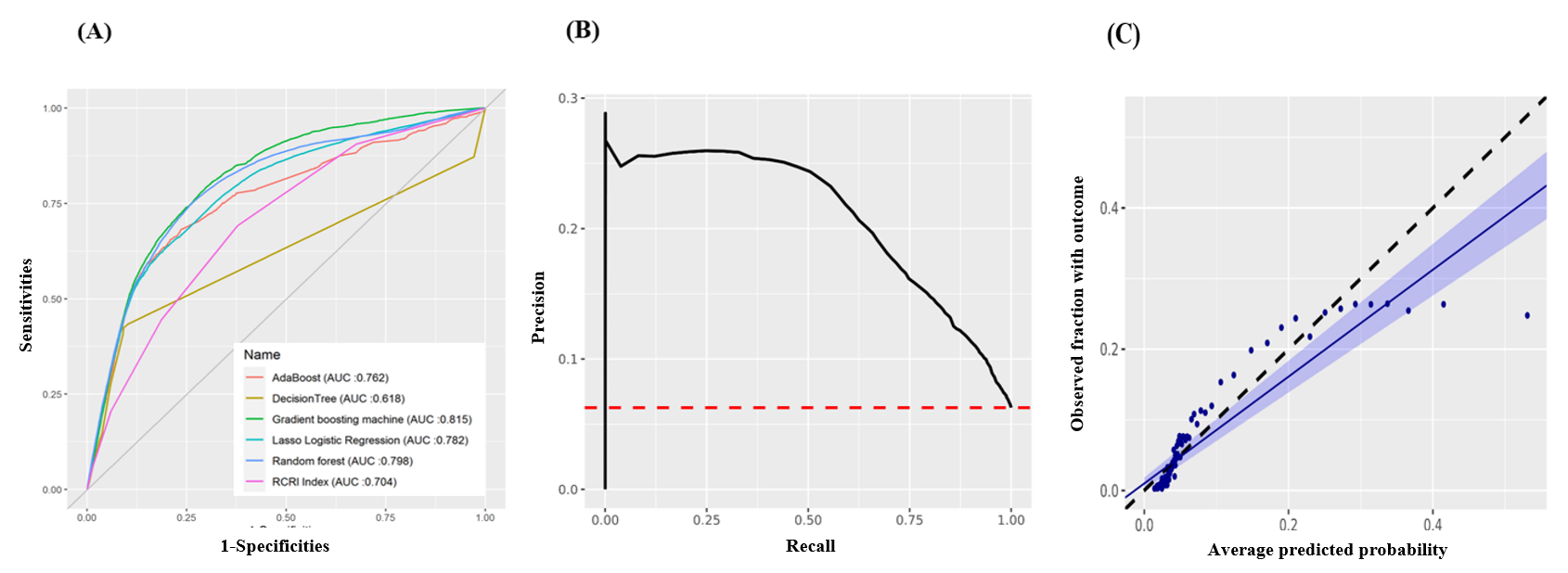


**Figure S2. Recombination prediction model (2) based on validation data**

(A) AUROC for predicting MACCE, demonstrating the model's ability to distinguish between patients with and without MACCE

(B) AUPRC for predicting MACCE, illustrating the precision-recall trade-off of the model (Gradient boosting machine).

(C) Calibration plot for MACCE, assessing the agreement between predicted probabilities and observed outcomes (Gradient boosting machine).


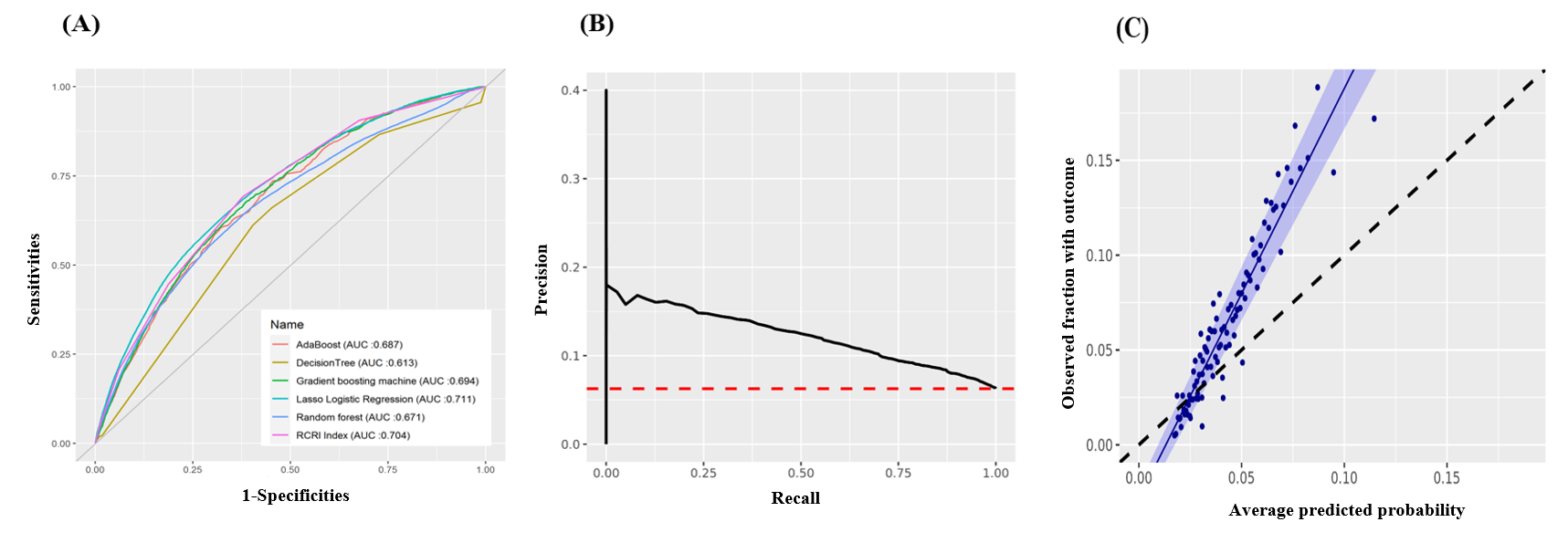


**Figure S3. Recombination prediction model (3) based on validation data**

(A) AUROC for predicting MACCE, demonstrating the model's ability to distinguish between patients with and without MACCE

(B) AUPRC for predicting MACCE, illustrating the precision-recall trade-off of the model (Lasso Logistic Regression).

(C) Calibration plot for MACCE, assessing the agreement between predicted probabilities and observed outcomes (Lasso Logistic Regression).
